# Root microbiota dynamics of perennial *Arabis alpina* are dependent on soil residence time but independent of flowering time

**DOI:** 10.1038/ismej.2016.109

**Published:** 2016-08-02

**Authors:** Nina Dombrowski, Klaus Schlaeppi, Matthew T Agler, Stéphane Hacquard, Eric Kemen, Ruben Garrido-Oter, Jörg Wunder, George Coupland, Paul Schulze-Lefert

**Affiliations:** 1 Department of Plant Microbe Interactions, Max Planck Institute for Plant Breeding Research, Cologne, Germany; 2 Plant–Soil-Interactions, Institute for Sustainability Sciences, Agroscope, Reckenholzstrasse 191, Zurich, Switzerland; 3 Cluster of Excellence on Plant Sciences (CEPLAS), Max Planck Institute for Plant Breeding Research, Cologne, Germany; 4 Department of Algorithmic Bioinformatics, Heinrich Heine University Düsseldorf, Düsseldorf, Germany; 5 Department of Plant Developmental Biology, Max Planck Institute for Plant Breeding Research, Cologne, Germany

## Abstract

Recent field and laboratory experiments with perennial *Boechera stricta* and annual *Arabidopsis thaliana* suggest that the root microbiota influences flowering time. Here we examined in long-term time-course experiments the bacterial root microbiota of the arctic-alpine perennial *Arabis alpina* in natural and controlled environments by 16S rRNA gene profiling. We identified soil type and residence time of plants in soil as major determinants explaining up to 15% of root microbiota variation, whereas environmental conditions and host genotype explain maximally 11% of variation. When grown in the same soil, the root microbiota composition of perennial *A. alpina* is largely similar to those of its annual relatives *A. thaliana* and *Cardamine hirsuta*. Non-flowering wild-type *A. alpina* and flowering *pep1* mutant plants assemble an essentially indistinguishable root microbiota, thereby uncoupling flowering time from plant residence time-dependent microbiota changes. This reveals the robustness of the root microbiota against the onset and perpetual flowering of *A. alpina*. Together with previous studies, this implies a model in which parts of the root microbiota modulate flowering time, whereas, after microbiota acquisition during vegetative growth, the established root-associated bacterial assemblage is structurally robust to perturbations caused by flowering and drastic changes in plant stature.

## Introduction

Plants host taxonomically structured bacterial consortia on and inside roots and leaves, designated the root and leaf microbiota (Vorholt, 2012; Bulgarelli *et al.*, 2013; Hacquard *et al.*, 2015). The start inoculum of the leaf-associated microbiota is derived from multiple sources, likely involving bacteria transmitted by aerosols, insects or soil particles (Vorholt, 2012; Bodenhausen *et al.*, 2013; Maignien *et al.*, 2014; Bai *et al.*, 2015). Conversely, root-associated bacterial consortia are mostly derived from the bacterial soil biome surrounding roots and establish rapidly. For example, a stable taxonomic structure in rice roots developed within 14 days after seed germination (Edwards *et al.*, 2015). Soil represents the most diverse ecosystem on earth with an exceptionally high bacterial species diversity that varies greatly between different soil types (Fierer and Jackson, 2006; Lauber *et al.*, 2009). Numerous studies employing next generation sequencing technologies have shown that soil type is a major determinant of root microbiota composition, most likely reflecting the different bacterial start inocula present in each soil type (Bulgarelli *et al.*, 2012; Lundberg *et al.*, 2012; Peiffer *et al.*, 2013; Schlaeppi *et al.*, 2014; Edwards *et al.*, 2015). Despite significant variation in microbiota composition at low taxonomic ranks, for example, genus- or species-level, a recent direct comparison of the root microbiota of eight flowering plant species, including monocots and dicots, revealed a co-occurrence of three main bacterial phyla comprising Actinobacteria, Bacteroidetes and Proteobacteria (Hacquard *et al.*, 2015). This finding suggests that the root microbiota and its overall taxonomic structure is a conserved plant trait across flowering plants.

An unresolved question in microbiota research is whether plant-associated bacterial assemblages contribute to the plasticity of complex plant traits. For example, field experiments with perennial *Boechera stricta* and laboratory experiments with annual *A. thaliana* have indicated that the bacterial root microbiota modulates flowering time (Wagner *et al.*, 2014; Panke-Buisse *et al.*, 2015). Conversely, root-associated bacterial consortia of *A. thaliana* appear to be affected by plant development stages and metatranscriptome studies revealed bacterial transcripts induced at bolting and flowering stages (Chaparro *et al.*, 2014).

Plant growth in the arctic-alpine environment requires an adaptation to a range of abiotic stresses, including water limitation, extreme temperature shifts and low nutrient availability (Billings and Mooney, 1968; Chapin, 1983; Chapin and Shaver, 1989; Margesin and Miteva, 2011). Root-associated bacterial members from three arctic-alpine plant species (*Oxyria digyna*, *Diapensia lapponica* and *Juncus trifidus*) appear to be enriched for potent microbial solubilizers of mineral phosphorus, a common but plant-inaccessible source of phosphorus in arctic-alpine environments (Nissinen *et al.*, 2012). This suggests the potential importance of microbiota members for plant growth and health in arctic-alpine environments (Richardson *et al.*, 2009). Perennial *Arabis alpina* is an arctic-alpine plant closely related to the annual *Arabidopsis thaliana*, which allows comparative studies of inter-species trait diversification such as flowering time (Beilstein *et al.*, 2010; Wang *et al.*, 2009). For example, the orthologue of the *A. thaliana* gene *FLOWERING LOCUS C* (*FLC*) that inhibits flowering until *A. thaliana* is exposed to winter temperature, was shown to be *A. alpina PEP1* (*PERPETUAL FLOWERING 1*), which limits flowering duration (Wang *et al.*, 2009). Perpetual flowering is characteristic for *A. alpina pep1* mutant plants, resulting in a drastic difference in plant stature due to the presence of reproductive shoots compared with wild-type (WT) plants (Wang *et al.*, 2009). Extensive allelic variation at *PEP1* also exists in natural populations, including loss of *PEP1* function alleles (Albani *et al.*, 2012). The adaptation of *A. alpina* to arctic-alpine environments, its perennial nature, the availability of genetic lines, as well as the close evolutionary relationship to *A. thaliana* make this plant species a suitable model to investigate the interplay of determinants for root microbiota composition and diversification.

Here we characterized *A. alpina* root-associated bacterial communities using several experimental approaches. We employed culture-independent community profiling by 16S rRNA gene sequencing to assess bacterial community composition of *A. alpina* plants grown in their natural habitat compared with plants grown under controlled environmental conditions in native or non-native soils (‘soil type and environment' experiment). We then investigated potential changes of the *A. alpina* root microbiota at two developmental stages (flowering vs vegetative) during an extended residence time of WT and *pep1* mutant plants in soil (‘time-course' experiment). Finally, we examined the diversification of the *A. alpina* bacterial microbiota by comparison with root microbial communities collected from two other Brassicaceae species, *A. thaliana* and *Cardamine hirsuta* (‘diversification' experiment).

## Materials and methods

### Soil types used and plant material

French soil was harvested in fall 2012 (‘FS Fall-12') at the Col du Galibier, France (45.061 N/6.402 E). Similar to earlier work (Bulgarelli *et al.*, 2012), Cologne soil batches were collected in fall 2010, in spring 2013 and in fall 2013 (termed ‘CS Fall-10', ‘CS Spring-13' and ‘CS Fall-13') at the Max Planck Institute for Plant Breeding Research in Cologne, Germany (50.958 N/6.856 E). Geochemical characterization of soil types was carried out by the 'Labor für Boden- und Umweltanalytik' (Eric Schweizer AG, Thun, Switzerland).

Three Brassicaceae plant species were investigated during this study. Experiments with *A. alpina* were conducted using the Spanish reference ecotype Pajares (Paj), the two French ecotypes F1-Gal5 and Gal60, as well as the mutant line *pep1* (Paj background; Wang *et al.*, 2009)*. A. alpina* Gal60 was harvested during the course of this study at the Col du Galibier, France. In addition, *A. thaliana* (ecotype Columbia, Col-0) and *C. hirsuta* (ecotype Oxford, Ox) were investigated.

### Plant growth

**Figure 1:**
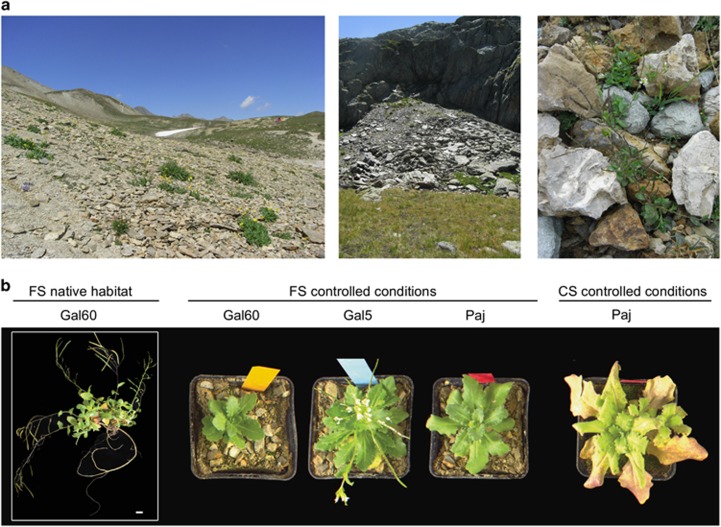
Growth phenotype of *A. alpina* in a natural French habitat and under controlled environmental conditions. (**a**) Natural habitat of *A. alpina* in the French Alps. (**b**) Growth morphology of different *A. alpina* accessions in a French soil (FS) in its native habitat in the French Alps, in FS under controlled environmental conditions and Cologne soil (CS) under controlled environmental conditions. Gal60, French *A. alpina* accession collected in its natural habitat in the French Alps, Col du Galibier; Gal5, French *A. alpina* accession; Paj, Spanish *A. alpina* accession.

We characterized *A. alpina* root-associated bacterial communities using three sets of experiments (Table 1, Supplementary Figure S1). First, for the ‘soil type and environment' experiment, individual flowering *A. alpina* plants of unknown age were excavated (designated Gal60) from their native habitat at the Col du Galibier (France) in fall 2012 to evaluate their natural root microbial communities (Figure 1a). To evaluate the effect of environmental factors on community assembly, we also collected soil from this site and transported it to Cologne to compare microbial communities from *A. alpina* Gal60 grown its native French soil in the natural environment with controlled environmental conditions in the greenhouse. To investigate possible host genotype-dependent effects on microbiota composition, we grew Gal60 alongside with a French genotype from a nearby location (Gal5) and a Spanish reference genotype (Paj) in the French soil in the greenhouse for 3 months. During harvest, Gal60 and Paj resided in the vegetative growth stage, while Gal5 plants started to flower (Figure 1b). Apart from their geographic origin, the three genotypes differ from each other in that the genotype Paj requires a vernalization treatment to flower, while Gal5 and Gal60 do not. In an additional subset of this experimental setup, we planted the *A. alpina* genotype Paj not only in French but also Cologne soil to study the effect of soil type on community composition. For the ‘soil type and environment' experiment conducted in the greenhouse, *A. alpina* seeds were surface-sterilized, sown onto the respective soil type, stratified for 4 days and a single plant per pot (at least four to five individuals per genotype) was grown for 3 months until harvest. In a second experimental setup, denoted ‘time course' experiment, individual *A. alpina* Paj plants were grown in two batches of Cologne soil, that is, two full factorial biological replicates, under controlled environmental conditions and harvested after 6, 12 and 28 weeks. Finally, in the ‘diversification' experiment, *A. alpina* (Paj), *C. hirsuta* (Oxford) and *A. thaliana* (Col-0) plants (four plants per pot) were grown in three batches of Cologne soil, that is, three full factorial biological replicates, under controlled environmental conditions for 6 weeks until harvest.

### 16S rRNA gene sample preparation, sequencing and analysis

We collected soil, rhizosphere and root compartments and prepared a DNA template for further processing using established protocols (Schlaeppi *et al.*, 2014). Briefly, amplicon libraries were prepared using the primers 799F (Chelius and Triplett, 2001) and 1193R (Bodenhausen *et al.*, 2013). Sequencing of samples with 454 pyrosequencing (Branford, CT, USA; ‘diversification' experiment) was conducted as previously described (Schlaeppi *et al.*, 2014), while the majority of the samples (‘soil type and environment' and ‘time course' experiment) were sequenced using Illumina (MiSeq) paired-end sequencing (San Diego, CA, USA; using a slightly modified PCR-amplification protocol, but targeting the same 16S rRNA gene region, see Supplementary Notes for details). We prepared a total of 201 DNA samples for sequencing: 59, 106 and 36 samples belonging to the ‘soil type and environment', ‘time course' and ‘diversification' experiment, respectively. In addition, 11 samples of the ‘diversification' experiment sequenced by the 454 technology, were re-sequenced using the Illumina protocol to investigate potential methodological biases (Table 1, Supplementary Figure S1). The bioinformatic analysis of sequence reads included de-multiplexing of samples using the QIIME software package (Caporaso *et al.*, 2010) and determination of operational taxonomic units (OTUs) on the three concatenated datasets using the UPARSE pipeline (Edgar, 2013). Afterwards, OTU tables were separated and investigated independently based on the three experimental setups (if not stated otherwise) and closer investigated by employing Principal Coordinate Analysis (PCoA) analyses and established linear model statistics using analysis of variance (ANOVA) and Bayesian model-based moderated *t*-tests to calculate differentially enriched OTUs (Bulgarelli *et al.*, 2012, 2015).

### Data deposition

Raw sequencing data were deposited in the NCBI Short Read Archive (SRA), BioProjectID PRJNA317760, accession number SRP073035. The reference numbers for the original raw sequencing data are SRR3350817 (L1151), SRR3351958 (L1264), SRR3353796 (L1291), L35 (SRR3355061), L905 (SRR3355062) and L1118 (SRR3355063). Custom R scripts can be found at http://www.mpipz.mpg.de/R_scripts. This provides a downloadable folder including the used R script, input data files and custom R-packages that were separated for the individual parts of the analysis.

## Results

### Marked bacterial community shifts in both *A. alpina* rhizosphere and root compartments

**Figure 2:**
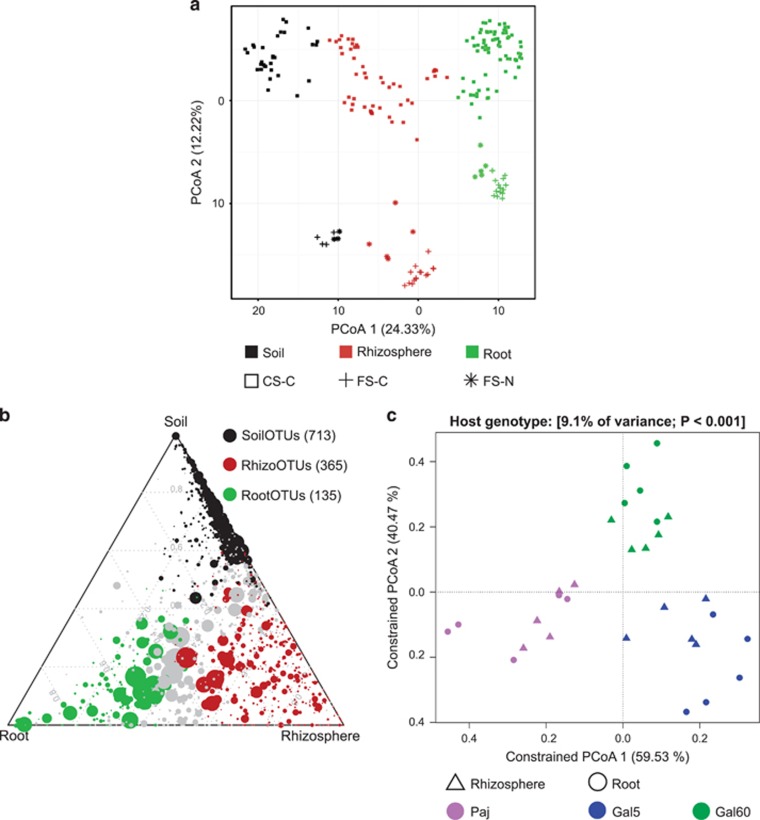
Bacterial community shifts in *A. alpina* rhizosphere and root compartments. (**a**) Unconstrained ordination revealing that most of the variation among all 201 samples from the 3 experimental setups is explained by the factor compartment (first principal coordinate axis) and soil type (second principal coordinate axis) based on the Bray–Curtis distance metric. (**b**) Ternary plot depicting the number of OTUs enriched in the soil, rhizosphere and root compartments of the 59 samples of the ‘soil type and environment' experiment (SoilOTUs (black), RhizoOTUs (brown), RootOTUs (green), respectively). Each circle depicts one individual OTU. The size of the circle reflects the relative abundance (RA). The position of each circle is determined by the contribution of the indicated compartments to the RA. Number in brackets: Enriched OTUs based on a Bayes moderated *t*-test; *P*<0.05 (FDR-corrected). (**c**) Constrained principal coordinates analysis on the genotypes Paj, Gal60 and Gal5 grown in the French Soil in the greenhouse using the Bray–Curtis dissimilarity and constraining by host genotype. In each case, the percentage of variation explained by each axis refers to the fraction of the total variance of the data explained by the constrained factor. CS-C, Cologne soil grown under controlled environmental conditions' FS-C, French soil grown under controlled environmental conditions; FS-N, French soil grown under native environmental conditions.

Plants were grown in microbe-containing soils and root, rhizosphere and soil compartments sampled as described previously (Figure 1; Bulgarelli *et al.*, 2012; Schlaeppi *et al.*, 2014). Briefly, the ‘soil compartment' refers to soil collected from unplanted pots and the ‘rhizosphere compartment' defines soil particles tightly adhering to roots that were collected by two washing steps. Washed roots were additionally sonicated to deplete bacterial epiphytes and enrich for root-inhabiting bacteria, designated ‘root compartment'. Bacterial community profiles for each compartment were generated by PCR amplification of the 16S rRNA gene by targeting region V5-V7 using PCR primers 799F (Chelius and Triplett, 2001) and 1193R (Bodenhausen *et al.*, 2013) followed by 454 or Illumina sequencing (Table 1 and Supplementary Information). We generated a total of 10 138 758 high-quality sequences from 201 samples (average of 50 342 and 2804-155 004 sequences per sample, individual read counts supplied in Supplementary Data S1). In addition, 11 samples of the ‘diversification' experiment that were sequenced with 454 were re-sequenced using the Illumina methodology and protocol, to estimate a potential methodological bias (533 801 total reads, Table 1, Supplementary Figure S1). The separation pattern between compartments was consistent between 454 and Illumina methodologies (Supplementary Figure S2) and we did not detect marked differences in taxonomic assignments (Supplementary Figure S3, see Supplementary Notes for details). This shows that the two sequencing technologies produce comparable results. For the 201 samples included in the main analysis of this study, we defined 8969 OTUs (⩾97% sequence similarity of 16S rRNA gene sequences), all belonging to the kingdom Bacteria. Across experiments, the tested compartment (soil, rhizosphere or root) explained 18–36% of community variation among samples (Figure 2a, Table 2). At phylum rank, unplanted soil communities were dominated with decreasing relative abundance by Proteobacteria, Actinobacteria and Gemmatimonadetes (Supplementary Figures S4–S7). In comparison to unplanted soil, root microbial communities displayed a significantly reduced alpha-diversity (Supplementary Figure S8, Supplementary Table S1) and were mainly inhabited by Proteobacteria, Actinobacteria and Bacteroidetes in all soil types tested. This co-occurrence of three bacterial phyla corresponds well with a recent assessment of the root microbiota of eight flowering plant species (Hacquard *et al.*, 2015). Taken together, the tested compartments had a pronounced effect on community structure, with the rhizosphere community displaying an alpha-diversity that is comparable to unplanted soil, whereas its taxonomic composition shared features with the soil and the highly distinctive root microbiota (Figure 2a, Supplementary Figures S4–S8).

### The *A. alpina* root microbiota is dependent on soil type but exhibits a converging taxonomic structure

We analyzed the 59 samples from the ‘soil type and environment' experiments separately, enabling us to dissect the relative contribution of the factors soil type, environment and host genotype on bacterial community composition (Table 1). We compared the taxonomic composition of bacterial assemblages in root and rhizosphere compartments with the soil biome of the *A. alpina* accession Gal60 in its native arctic-alpine environment at the Col du Galibier (France, Figure 1a). In addition, the Gal60 accession was grown in the same native soil, but under controlled environmental conditions, together with the accessions Gal5 and Paj that are derived from a different site at the Col du Galibier and the Cordillera Cantábrica in Spain, respectively. In a further subset of this experiment, the accession Paj was grown under controlled environmental conditions in Cologne soil (Figure 1b, Supplementary Tables S3 and S4).

To quantify the contribution of individual factors to the observed variation in community profiles, we used Bray–Curtis (accounting for OTU abundances but not phylogenetic distances), unweighted (accounting for phylogenetic distances but not abundances) and weighted (accounting for OTU abundances and phylogenetic distances) UniFrac distance metrics as β-diversity (between-sample diversity) estimates (Lozupone and Knight, 2005). A constrained Canonical Analysis of Principal coordinates (CAP), followed by a permutation-based ANOVA (PERMANOVA, Table 2, Figure 2c,Supplementary Figure S9) revealed that the factor soil type explained 11–15% of the observed variation, followed by environment (8–11%) and host genotype (5–12%). Both soil type and environment significantly influenced community composition, and we found no significant interaction between the soil type and environment variables (Supplementary Figure S9a). Notably, host genotype affected community composition with a higher significance using weighted compared with unweighted UniFrac, suggesting that host genotype preferentially acts on abundant community members. At the time of harvest, the accession Gal5 was flowering while Gal60 and Paj resided in the vegetative stage (Figure 1b). Because only Paj requires a vernalization treatment to flower (Gal5 and Gal60 do not), the differences in developmental stages could potentially confound the observed host genotype-dependent effects (see below). In addition, the three genotypes were only grown in one soil type; therefore, we cannot make conclusions on potential environment-specific adaptations of individual genotypes. The impact of soil type on community profiles was apparent at the highest taxonomic rank. For example, differential enrichments of the phyla Actinobacteria and Firmicutes were found in French and Cologne soils (Supplementary Table S5 and Supplementary Figure S4). In addition, soil type-dependent differences of the bacterial soil biome at phylum rank converged progressively towards more soil type-independent community profiles in rhizosphere and root compartments (Supplementary Table S5), indicating that roots provide a niche for the assembly of a stable bacterial consortium despite contrasting soil types and environments.

Next, we quantified the number of OTUs that were enriched in a compartment across soil types and environments (Supplementary Information; Bulgarelli *et al.*, 2012). We refer to OTUs significantly enriched in soil, rhizosphere and root compartments as SoilOTUs, RhizoOTUs and RootOTUs, respectively. Of a total of 1676 OTUs, the compartment-enriched OTUs gradually declined from 713 SoilOTUs to 365 RhizoOTUs and 135 RootOTUs, with the remaining 463 OTUs being shared among compartments (Figure 2b). Of the RootOTUs, ~30% were shared across all tested soil types and environmental conditions (Supplementary Figure S10). Taken together, this analysis shows that *A. alpina* roots accommodate a taxonomically congruent bacterial community in contrasting environments and produce a marked rhizosphere effect, as evidenced by the identification of 365 RhizoOTUs.

### The *A. alpina* root microbiota is dependent on soil residence time and independent of flowering time

To evaluate potential changes of *A. alpina* root- and rhizosphere-associated microbiota composition over time, *A. alpina* samples were collected at 6, 12 and 28 weeks after sowing in Cologne soil under controlled environmental conditions (Table 1,Supplementary Information). In the context of this ‘time-course' experiment we also investigated whether host developmental stage (non-flowering and flowering) affects bacterial community structure. We planted the WT *A. alpina* accession Paj together with the perpetually flowering mutant *pep1* (Wang *et al.*, 2009). This mutant started to flower after 12 weeks, whereas WT Paj plants remained in the vegetative stage until the end of the experiment.

**Figure 3:**
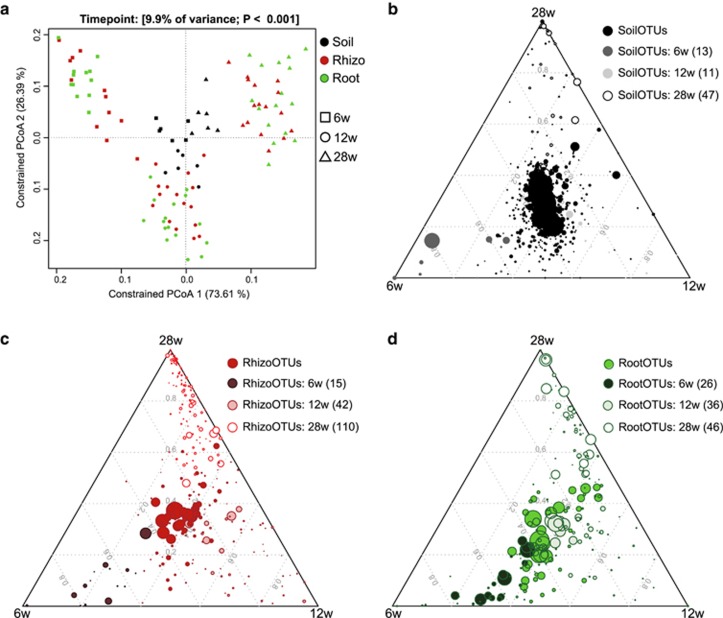
*A. alpina* root-associated bacterial communities depend on soil residence time. (**a**) Principal coordinate analysis on samples from the ‘time course' experiment (106 samples) constrained for the factor time point for soil, rhizosphere and root compartments at 6, 12 and 28 weeks, respectively. The percentage of variation explained by each axis refers to the fraction of the total variance of the data explained by the constrained factor. (**b**–**d**) Soil-, rhizosphere- and root-enriched OTUs (RootOTUs, RhizoOTUs, SoilOTUs) including OTUs observed across all tested time points and OTUs specific for individual time points. Time point-specific SoilOTUs (**b**), RhizoOTUs (**c**) and RootOTUs (**d**). The mean of soil (**b**), rhizosphere (**c**) or root (**d**) samples at each time point is plotted. Number in brackets: total number of OTUs enriched in the respective compartment or time point. Enriched OTUs are based on a Bayes moderated *t*-test; *P*<0.05 (FDR-corrected).

**Figure 4:**
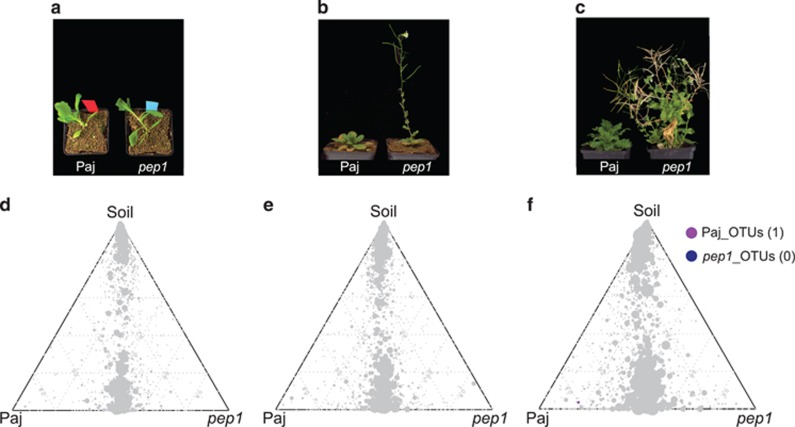
The *A. alpina* root microbiota is independent of flowering time. (**a**–**c**) Growth morphology of *A. alpina* wild-type (Paj) and *pep1* mutant plants across the ‘time course' experiment. (**d**–**f**) Ternary plots of OTUs enriched in the ‘time course' experiment across the soil and root compartments of the *A. alpina* wild-type (Paj) and *pep1* mutant plants after 6 weeks (**a**, **d**), 12 weeks (**b**, **e**) and 28 weeks (**c**, **f**). Each circle depicts one individual OTU. The size of the circle reflects the relative abundance (RA). The position of each circle is determined by the contribution of the indicated compartments to the RA. Number in brackets: total number of OTUs enriched in the respective plant line. OTUs enriched in the two plant lines are based on a Bayes moderated *t*-test; *P*<0.05 (FDR-corrected).

Within this experimental setup, the factor time point explained 7–12% of the observed variation (Table 2, Figure 3a). Considering the interaction between time point and compartment, we found a strong interaction between those two factors, explaining 11% of the variance of the data (Supplementary Figure S9b). Interestingly, only root and rhizosphere community profiles were affected over time, while the initial bacterial community present in unplanted soil remained largely stable over the tested time frame from 6 to 28 weeks across the used soil batches (Figure 3, Supplementary Figure S5). The stability of soil samples over time was apparent since these samples did not separate within the PCoA and most SoilOTUs remained in the center of the ternary plot, reporting their equal abundance across all tested time points. The observed strong impact of residence time of plants in soil on bacterial root and rhizosphere communities was apparent at high taxonomic rank, for example, by an increase in the relative abundance of Proteobacteria and decrease of Bacteroidetes in roots over time (Supplementary Figure S5 and Supplementary Table S6). To identify OTUs that explain these time point-dependent changes, we first determined all SoilOTUs (745), RhizoOTUs (271) and RootOTUs (200). Around 62% of RhizoOTUs and 54% RootOTUs significantly changed in relative abundance over time, whereas this was only the case for ~10% of SoilOTUs (Figures 3b–d). Closer inspection showed that changes in rhizosphere and root microbiota profiles were characterized by a continuous increase in the total number of Rhizo- and RootOTUs over time (Figures 3c and d). Remarkably, despite drastic differences in plant stature, we did not detect significant changes between the root and rhizosphere microbiota of WT and *pep1* mutant plants (Table 2, Figure 4). None of the Root- or RhizoOTUs differentially accumulated in 6- or 12-week-old WT and *pep1* plants, when 12-week-old *pep1* plants were flowering (Figures 4d and e). A single low-abundant OTU (OTU927, order Myxococcales) was enriched in 28-week-old WT plants when *pep1* plants were flowering and WT plants remained in the vegetative phase (Figure 4f). In addition, we detected stable nutrient contents and soil parameters in unplanted soil compared with soil samples recovered from pots containing 28-week-old plants (Supplementary Tables S3 and S4). In summary, our findings reveal marked residence-time-dependent changes of *A. alpina* root- and rhizosphere-associated bacterial consortia, but a stability of the root microbiota against the onset and perpetual flowering of *A. alpina* and concomitant drastic changes in plant stature.

### The composition of the root microbiota of perennial *A. alpina* is similar compared with the annuals *A. thaliana* and *C. hirsuta*

**Figure 5:**
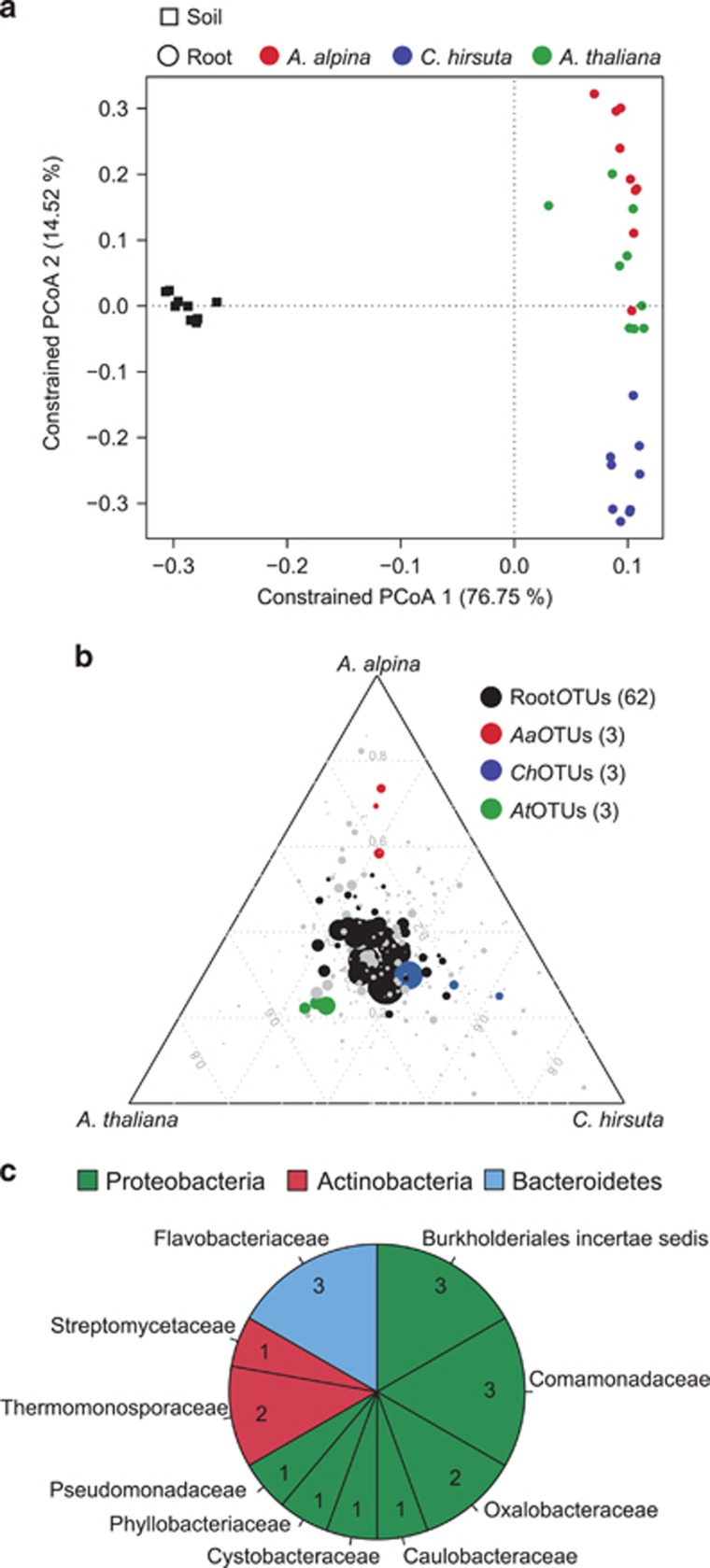
The bacterial root microbiota of *A. alpina* is similar compared with *A. thaliana* and *Cardamine hirsuta*. (**a**) Constrained principal coordinates analysis (PCoA) based on the Bray–Curtis distances on the 36 samples of the ‘diversification' experiment constrained by sample groups. (**b**) OTUs enriched on roots of *A. alpina*, *C. hirsuta* and *A. thaliana*. Color-coded in black are root-enriched OTUs (RootOTUs). All enriched OTUs are based on a Bayes moderated *t*-test, *P*<0.05 (FDR-corrected). Number in brackets: total number of OTUs enriched in the respective plant species. (**c**) Pie chart reporting family distribution of 18 shared OTUs based on parametric Tukey's honest significant difference and Bayesian and non-parametric Mann-Whitney statistics.

Next, we planted perennial *A. alpina* side-by-side with the annuals *C. hirsuta and A. thaliana* in Cologne soil for 6 weeks to examine potential diversification of the corresponding root microbiota (Table 1, Supplementary Figures S11a and b and Supplementary Information). We found evidence for host species-specific root microbiota profiles in which the factor host species explained 7–10% of the observed variation (Table 2). PCoA, constrained by host species, revealed that samples from *A. alpina* roots clustered closely together with those of *A. thaliana* although these plant species diverged ~45 Myr ago (Figure 5,Supplementary Figure S11a; Beilstein *et al.*, 2010). Root samples of *C. hirsuta* clustered separately from the former two hosts although *A. alpina* diverged from *C. hirsuta* only ~10 Myr ago (Figure 5a). Thus, for the tested plant species host divergence time appears to be incongruent with root microbiota diversification. The impact of host species on bacterial root communities was detectable at phylum rank by a significant reduction in the relative abundance of Bacteroidetes in root samples of *A. alpina* (Supplementary Figure S7a). In addition, significant differences were detected for one, three and five bacterial families in *A. thaliana*, *C. hirsuta* and *A. alpina*, respectively (Supplementary Figure S7b,Supplementary Table S7). Each tested host species was also enriched for a few species-characteristic OTUs that displayed quantitative differences in relative abundance (designated *At*OTUs, *Ch*OTUs and *Aa*OTUs; Figure 5b). However, the majority of RootOTUs (62) were similarly detected in roots of all three host species (Figure 5b). In addition, a total of 18 RootOTUs were significantly enriched in roots of all 3 plant species across the tested soil batches (Figure 5c,Supplementary Figure S12a). These shared OTUs were barely detectable in unplanted soil and together represented almost 50% of the root-associated consortia (Supplementary Figure S12b). The shared OTUs belonged to 3 phyla, with 12 OTUs assigned to Proteobacteria and 3 each to Actinobacteria and Bacteroidetes (Figure 5c). In summary, we found few host species-characteristic OTUs, while the majority of the root microbiota was shared among the tested host species.

## Discussion

Here, we quantified the relative contributions of factors explaining variation in *A. alpina* root-associated bacterial consortia by analyzing high-quality 16S rRNA reads from >200 samples and multiple environmental factors. This revealed the variable compartment as strongest determinant of community variation (18–36%). Additional variables influencing community variation were soil type (11–15%), residence time of plants in soil (7–12%), environmental conditions (8–11%), host species (7–10%) and host genotype (5–12%). The relative contributions of the factors compartment, soil type and host species in this study are comparable to an earlier report on root microbiota diversification between *A. thaliana* relatives (Schlaeppi *et al.*, 2014). Despite the observed host species-dependent differences in root microbiota profiles, we identified a largely shared bacterial assemblage whose taxonomic structure is similar to the microbiota intersection between *A. thaliana*, *A. halleri*, *A. lyrata* and *C. hirsuta* (Schlaeppi *et al.*, 2014). This points to potentially common services provided by these bacterial taxa for plant growth and health across five tested Brassicaceae host species. However, in contrast to previous work showing only weakly differentiated bacterial rhizosphere assemblages compared with unplanted soil biomes in annual *A. thaliana* and *C. hirsuta* plants that were partially performed in the same soil type and growth conditions (Bulgarelli *et al.*, 2012; Lundberg *et al.*, 2012; Schlaeppi *et al.*, 2014), we detected a pronounced rhizosphere effect for *A. alpina* at all tested time points and across soil types and environments. This suggests that within the Brassicaceae, evolutionary diversification has apparently produced host species-specific differences in bacterial community differentiation in the rhizosphere compartment despite an overall convergent community composition inside roots (Bulgarelli *et al.*, 2012; Lundberg *et al.*, 2012; Schlaeppi *et al.*, 2014). This striking feature of *A. alpina* may be due to its phylogenetic distance to *A. thaliana* and *C. hirsuta*, adaptation to the arctic-alpine habitat or its perennial lifestyle.

Based on similar CAP analyses, the variation of root microbiota profiles explained by intra-species genetic diversity in *A. alpina* (7–10%) is remarkably similar to those reported for corn (5–8% *Zea mays*), barley (~6% *Hordeum vulgare*) and rice (3–5% *Oryza sativa*), suggesting that host genotype determines a small, but significant amount of microbiota variation across all tested flowering plants (Peiffer *et al.*, 2013; Bulgarelli *et al.*, 2015; Edwards *et al.*, 2015). In addition, our study supports and extends earlier observations that the Brassicaceae host phylogeny is incongruent with root microbiota diversification (Schlaeppi *et al.*, 2014), suggesting that host and root microbiota structure are subject to independent diversification processes. However, our experimental approach does not allow us to assess whether inter- exceeds intra-species root microbiota diversification, as this requires information on population structure and genetic diversity of each host and sampling in more diverse environments. This experimental limitation does not permit us to completely exclude correlated evolutionary processes driving host and microbiota diversification.

Previous work with *A. thaliana* suggested that the root-associated bacterial assemblage is affected by plant development, showing differences in community structure between seedling and vegetative stage and bacterial transcripts induced at bolting and flowering stages (Chaparro *et al.*, 2014). However, these experiments were conducted with WT *A. thaliana* only and do not exclude the possibility that changes in root microbiota structure or activity are influenced by differences in the residence time of the plants in soil. While we cannot exclude differences in root microbiota activity, bacterial community structure on WT *A. alpina* and *pep1* mutant plants was essentially indistinguishable throughout the analyzed time course, even though the *pep1* mutant started to flower during the analysis whereas the WT did not. This strong, genetically determined difference in flowering time enabled us to uncouple flowering from plant residence time-dependent changes in the microbiota. This strongly suggests that the bacterial root assemblage, once established during the vegetative plant growth phase, is structurally robust to the onset and perpetual flowering of *A. alpina*. During the transition to flowering, the shoot apex becomes a sugar sink (Bernier, 1988) and such changes might reduce photosynthates available for transport to the roots. In addition, enhanced root secretion of defense-related proteins has been reported during flowering of *A. thaliana* (De-la-Peña *et al.*, 2010). Such metabolic changes do not detectably affect the *A. alpina* root microbiota as evidenced by an indistinguishable root-associated bacterial community composition of non-flowering WT and flowering *pep1* mutant plants. Conversely, differences in soil bacteria between soil types can influence flowering time as recently shown for perennial *B. stricta* and annual *A. thaliana* although the underlying mechanisms and specific bacterial taxa causing these flowering time shifts remain unidentified (Wagner *et al.*, 2014; Panke-Buisse *et al.*, 2015). Collectively, this suggests a unidirectional rather than bidirectional interference model in which particular root microbiota members modulate flowering time, whereas the established root-associated microbial community remains robust to perturbations caused by flowering onset and drastic changes in plant stature. This stability of root-associated bacterial assemblages against changes in host developmental stage or physiological status is supported by the finding that community profiles of *A. thaliana* roots at bolting and leaf senescence stage were indistinguishable (Lundberg *et al.*, 2012). Acquisition of the root microbiota in rice plants was shown to initiate within 24 h and approach steady-state within 14 days (Edwards *et al.*, 2015). Thus, it is possible that after microbiota acquisition at the seedling stage, microbe–microbe interactions stabilize the root-associated community composition, thereby rendering the assemblage resistant to flowering time-associated metabolic changes in the host.

We monitored microbiota profiles of *A. alpina* roots for up to 7 months and found marked changes over time at all tested taxonomic ranks (7–12% of variation) that are dependent on the residence time of plants in soil rather than plant developmental stage or plant stature. These long-term dynamics of the *A. alpina* root microbiota contrasts with the stability of the bacterial community in unplanted soil, monitored in parallel over the same time period. Comparable long-term time-course experiments are not possible with short-lived annual *A. thaliana* and time-course experiments covering most of the life cycle of other perennial or long-lived annual plants are, to our knowledge, currently unavailable. Given the robustness of the *A. alpina* root-associated bacterial assemblage to flowering onset and changes in plant stature (see above), we speculate that the observed long-term root microbiota dynamics are a consequence of competition among bacteria rather than altered host-bacteria interactions. Alternatively, unknown host factors linked to the longevity of *A. alpina* such as a local depletion of soil-borne mineral micronutrients due to prolonged plant growth might contribute to the observed microbiota dynamics. It will be interesting to investigate in future work whether other perennial plant species exhibit a similar soil residence time-dependent dynamics of the root microbiota and whether the observed community shift is linked to altered community functions.

## Figures and Tables

**Table 1:** Experimental design and numbers of replicates per DNA sample across experimental setups

*Compartment*	*Host plant*	*Soil type and environment*			*Time course*						*Diversification*			*454 vs Illumina*
		*CS_C*	*FS_C*	*FS_N*	*6 weeks*		*12 weeks*		*28 weeks*					
					*rep1*	*rep2*	*rep1*	*rep2*	*rep1*	*rep2*	*rep1*	*rep2*^a^	*rep3*	*rep2*^a^
Soil	—	4	4	3	3	3	3	3	3	3	3	3	3	3
Rhizosphere	*A. alpina* (Paj)	4	5	—	3	3	4	4	4	4	—	—	—	—
	*A. alpina* (*pep1*)	—	—	—	3	3	4	4	4	4	—	—	—	—
	*A. alpina* (Gal5)	—	5	—	—	—	—	—	—	—	—	—	—	—
	*A. alpina* (Gal60)	—	5	5	—	—	—	—	—	—	—	—	—	—
Root	*A. thaliana* (Col-0)	—	—	—	—	—	—	—	—	—	3	3	3	3
	*C. hirsuta* (Ox)	—	—	—	—	—	—	—	—	—	3	3	3	2
	*A. alpina* (Paj)	4	5	—	3	3	4	4	4	4	3	3	3	3
	*A. alpina* (*pep1*)	—	—	—	3	3	4	4	4	4	—	—	—	—
	*A. alpina* (Gal5)	—	5	—	—	—	—	—	—	—	—	—	—	—
	*A. alpina* (Gal60)	—	5	5	—	—	—	—	—	—	—	—	—	—
	Soil type	Cologne	France		Cologne						Cologne			Cologne
	Residence time	12 weeks	12 weeks	ND	6 weeks	6 weeks	12 weeks	12 weeks	28 weeks	28 weeks	6 weeks	6 weeks	6 weeks	6 weeks
	Harvest date soil	Spring13	Fall12		Spring13	Fall13	Spring13	Fall13	Spring13	Fall13	Fall10	Spring13	Fall13	Spring13
	Environment	Controlled		Native	Controlled						Controlled			Controlled
	Seq. technology	Illumina			Illumina						454			Illumina

Abbreviations: CS, Cologne soil; FS, French soil; ND, not determined.

Experimental setup comparing bacterial community composition under natural growth conditions (‘soil type and environment' experiments), prolonged residence time of plants in soil (‘time-course' experiment) on three different plant species (‘diversification' experiment). The first two columns characterize the tested compartment, host plant and plant genotype. The numbers indicate the number of sequenced DNA samples. ‘Soil type and environment' experiment: plants were grown in CS or FS under controlled environmental conditions (C) or in their native habitat in France (N). ‘Time course' experiment: *A. alpina* plants were grown under controlled environmental conditions in CS for 6, 12 and 28 weeks. ‘Diversification' experiment: *A. alpina*, *C. hirsuta* and *A. thaliana* were grown under controlled environmental conditions in CS for 6 weeks. Indicated are the number of independent biological replicates (rep1–rep3), the soil type used for growing plants (see also Supplementary Tables S3 and S4), the harvest date of the soil, the environmental conditions used for plant growth and the employed sequencing technology. ND, not determined.

a Represents the same DNA samples that were processed with both 454 and Illumina sequencing technologies.

**Table 2:** Determining drivers of bacterial community assembly using CAP

	*Constrained factor*	*Bray–Curtis*			*Weighted Unifrac*			*Unweighted Unifrac*		
		*%*	P*-value*	*CI*	*%*	P*-value*	*CI*	*%*	P-*value*	*CI*
Soil type+environment	Compartment^a^	31	***	21, 48	33	***	20, 52	36	***	23, 64
	Environment^a^	11	***	8, 16	8.2	***	5, 13	9.8	***	7, 14
	Compartment^b^	25	***	15, 47	24	***	13, 46	37	***	19, 78
	Genotype^b^	9.1	***	7.3, 11	12	***	8.5, 16	4.8	*	4.2, 5.5
	Compartment^c^	31	***	20, 50	39	***	22, 67	33	***	21, 53
	Soil type^c^	15	***	9, 26	11	***	6, 20	15	***	10, 25
Time course	Compartment	19	***	14, 27	25	***	17, 39	18	***	14, 26
	Time point	9.9	***	7.8, 13	12	***	8.5, 18	6.6	***	5.7, 7.8
	Soil batch	6.6	***	4.9, 9.1	5.3	***	3.6, 8	8.4	***	6.6, 11
	Flowering stage	1.5	NS	1.2, 1.9	1.3	NS	1.0, 1.9	1.8	*	1.3, 2.5
	Mutant background	0.6	NS	0.4, 0.8	0.8	NS	0.5, 1.2	0.7	NS	0.5, 1.1
Diversification	Compartment	21	***	12, 37	26	***	12, 51	18	***	12, 31
	Soil batch	19	***	14, 28	20	***	12, 32	18	***	14, 24
	Plant species	10	***	8, 14	10	***	7, 15	7.3	***	6, 9

Abbreviations: CAP, constrained analysis of principal coordinates; CI, confidence interval; NS, non-significant; PERMANOVA, permutation-based analysis of variance.

Variation (in %) between samples in the ‘soil type and environment', ‘time course' and ‘diversification' experiments based on Bray–Curtis, weighted and unweighted UniFrac distances, constraining for the indicated factors. *P*-value based on PERMANOVA (999 permutations). *P<0.05, **P<0.01, ***P<0.001. Samples were analyzed separately according to the three different experimental setups as stated in Table 1. In addition, the ‘soil type and environment' samples were separated as follows:

a Gal60 grown at the natural site and the greenhouse.

b Gal60, Gal5 and Paj grown in French soil in the greenhouse.

c Paj grown in the French and Cologne soil in the greenhouse.
