# Supplementary material for: Root microbiota dynamics of perennial Arabis alpina are dependent on soil residence time but independent of flowering time
Source: ISME J. 2016 Aug 2;11(1):43–55. doi: 10.1038/ismej.2016.109 (PMC5097464; doi:10.1038/ismej.2016.109)
Supplement: Supplementary Methods [file ismej2016109x1.docx]

# Supplementary Methods

### Storage and preparation of soil material

Soil types used in this study were collected at the following locations: ‘CS Fall-10’, ‘CS Spring-13’ and ‘CS Fall-13’ (50.958 N/ 6.856 E, Cologne, Germany), and ‘FS Fall-12’ (45.061 N/ 6.402 E, Col du Galibier, France). Soil types were harvested at the following dates: ‘CS Fall-10’ in September 2010, ‘CS Spring-13’ in March 2013, ‘CS Fall-13’ in September 2013 and ‘FS Fall-12’ in August 2012. At all locations soil was not exposed to agricultural use for several years. To excavate material the topsoil (10-15 cm) was removed and a layer between -15 and -30 cm collected, followed by storage of soil for one week at ambient temperature for drying (according to Bulgarelli *et al.*, 2012). Homogenized soil was stored at 4°C until further usage. Upon usage, soil samples were sieved with a 5 mm followed by a 2 mm mesh and afterwards distributed to 7x7x9 cm or 9x9x9 cm pots for experiments. Geochemical characterization was carried out by the “Labor für Boden- und Umweltanalytik” (Eric Schweizer AG, Thun, Switzerland) and results are listed in **Supplementary Tables S3-S4**. For the *A. alpina* time course experiment ‘CS Spring-13’ or ‘CS Fall-13’ soil was mixed in a 1:1 ratio with autoclaved sand (at 121 °C for 20 min, stored overnight, followed by a second autoclaving under the same conditions) to ensure a sufficient aeration of plant roots for a growth period of 7 months. This was concluded from pilot experiments in which *A. alpina* plants, cultured in unsupplemented Cologne soil, were found to grow poorly (data not shown). This growth phenotype is likely due to an adaptation of *A. alpina* to the natural sandy loam of the arctic-alpine soil (**Supplementary Table S3**). In all other cases, soil was used without further amendments according to previous protocols (Bulgarelli *et al.*, 2012).

### Plant material

Three plant species belonging to the Brassicaceae lineage were investigated during this study, namely *Arabis alpina,* *Cardamine hirsuta* (accession Oxford, Ox) and *Arabidopsis thaliana* (accession Columbia, Col-0, **Supplementary Figure S11a,b**). For *A. alpina* the following ecotypes were used: Spanish accession Pajares (Paj), the two French accessions F1-Gal5, Gal60 and the mutant line *pep1* (in Paj background, **Figure 1b**; Wang et al., 2009). Seed material for *A. thaliana*and *C. hirsuta* was kindly provided by Dr. Klaus Schläppi (Agroscope Institute for Sustainability Sciences ISS). Seed material from *A. alpina* was supplied by Dr. Maria Albani and Dr. Jörg Wunder (Department of Plant Developmental Biology, Max Planck Institute for Plant Breeding Research). *A. alpina*accession Gal60 was collected at the natural site in France at the Col du Galibier together with the respective natural soil.

### Plant growth conditions

To assess bacterial community structure for the ‘soil type and environment’ experiments two approaches were taken. First, *A. alpina* plants were directly collected in the French Alps (45.061 N/ 6.402 E, Col du Galibier, France; i.e. ‘natural site’ experiments) and, second, soil from the same location was excavated and used for plant growth under greenhouse conditions (i.e. ‘natural soil’ experiments). For the ‘natural environment’ experiment healthy-looking flowering plants were excavated including at least 10 cm of the main root. Excavated plants were transferred to pots including natural soil and transported to Cologne, Germany. Plants were kept for approximately one day at 4°C until further processing. Additionally, *A. alpina* seeds were collected at the same location (designated *A. alpina* accession Gal60). For the ‘natural soil’ experiments, sterilized seeds of the *A. alpina* accessions Paj, Gal5 and Gal60 were directly sown onto French soil at a density of one plant per pot (9x9 cm pots) and stratified for three to four days. Plants were grown in the greenhouse for three months under long day conditions (16/8 hours day/night with a temperature of 22°C/18°C and a relative humidity of 70%). At the time of harvest, Paj and Gal60 plants were in the vegetative growth stage whilst Gal5 plants were flowering. For comparison of this experiment with the ‘time course’ experiment (see below), the *A. alpina*accession Paj was also grown for three months in ‘CS Fall-10’. For bulk soil samples, unplanted pots were kept under the same conditions as pots containing plants until harvest. For every condition, three to five independent DNA samples, i.e. dependent biological replicates, were included in the analysis (**Table 1**).

For the *A. alpina* ‘time course’ experiment surface-sterilized seeds were sown at a density of one plant per pot (9x9 cm pots) and stratified for three to four days. Plants were grown in the greenhouse for up to 28 weeks under long day conditions (16/8 hours day/night with a temperature of 22°C/18°C and a relative humidity of 70%). The root, rhizosphere and soil compartments were harvested 6, 12 and 28 weeks after sowing. After 6 weeks of sowing, 6-8 pots were pooled per replicate during harvest, whereas after 12 and 28 weeks one pot per replicate was used for further DNA isolation. For every condition three to four DNA samples per biological replicate were included in the analysis. In total, two fully independent biological replicates were conducted using the soil batches ‘CS Spring-13’ and ‘CS Fall‑13’.

For the ‘diversification’ experiment surface sterilized seeds were sown at a density of four plants per pot (7x7 cm pots) and stratified for three to four days. Plants were grown in the greenhouse for 6 weeks under short day conditions (8/16 hours day/night with a temperature of 22°C/18°C and a relative humidity of 70%). After 6 weeks all plants were in the vegetative growth stage. For each sample three to four pots, containing a total of 12-16 plants, were pooled during harvest. For every species three independent DNA samples per condition were included in the analysis. In total three independent biological replicates were conducted using the soil batches ‘CS Fall-10’, ‘CS Spring-13’ and ‘CS Fall-13’.

### Sampling of DNA from the root, soil and rhizosphere compartment

After harvesting, roots were mechanically separated from the adhering soil particles and a defined root segment of 3 cm starting 0.5 cm distant from the hypocotyl was sampled for the comparison of microbiota profiles of *A. alpina*, *C. hirsuta* and *A. thaliana*. For the ‘time course’ and ‘soil type and environment’ experiment a segment of 4.5 cm was collected. Soil particles still attached to the roots were removed by gentle tapping. Roots were collected in 15 ml falcons containing 5 ml phosphorus buffered saline (PBS)-S buffer (130 mM NaCl, 7 mM Na_2_HPO_4_, 3 mM NaH_2_PO_4_, pH 7.0, 0.02% Silwet L-77) and washed for 15 minutes at 180 rpm on a shaking platform. Roots were transferred to a new falcon and the remaining soil particles were centrifuged for 20 minutes at 15 000 x g and the pellet collected in liquid nitrogen and stored at -80°C. This fraction represents the rhizosphere compartment. After a second washing of the root samples, roots were transferred to a new falcon tube and sonicated 10 times at 160 W with 30 second brakes (Bioruptor Next Gen UCD-300, Diagenode, Liège, Belgium). To validate that the sonication step effectively removed the rhizoderm of *A. thaliana*, *C. hirsuta* and *A. alpina*, scanning electron microscopy (SEM) pictures were taken from individual root samples after sonication (**Supplementary Figure S11c,d**). Roots were transferred to fresh PBS-S, briefly dried on Whatman® glass microfiber filters (Sigma-Aldrich, Deisenhofen, Germany), transferred to 2 ml tubes and frozen in liquid nitrogen for further storage at -80°C. Unplanted soil samples were collected from an unplanted pot after removing 0.5 cm of the top soil, immediately frozen in liquid nitrogen and stored at -80°C. For DNA isolation the FastDNA ® SPIN for soil kit (MP Biomedicals, Solon, USA) was utilized. Before, DNA isolation, samples were homogenized using the Precellys®24 tissue lyzer (Bertin Technologies, Montigny-le-Bretonneux, France) at 6 500 rpm for 30 seconds. Root samples were homogenized three times with repeated freezing in liquid nitrogen. Rhizosphere and soil samples were homogenized once. Thereafter, DNA was extracted using the FastDNA ® SPIN for soil kit according to the manufacturer’s protocol.

# Generation of 16S rRNA gene amplicon libraries

### 454 Pyrosequencing

DNA concentration was assessed using the Quant-iT™ PicoGreen dsDNA assay kit (Life Technologies, Darmstadt, Germany). 40 µl of a 1:200 dilution of PicoGreen was added to 4 µl of DNA in a 96 well plate. For calculation of the DNA concentration, a dilution series of standard lambda DNA ranging from 0.5 to 20 ng/µL was included on the same plate. Fluorescence was measured using the IQ5 real-time PCR Thermocycler (Biorad, Munich, Germany; 30 sec at 25°C, 3x30 seconds at 25°C for measuring fluorescence, 30 seconds at 15°C). Thereafter, DNA was adjusted to a final concentration of 3.5 ng/µl. A PCR amplicon library was generated using the primers 799F and 1193R spanning ~400 bp of the hypervariable region V5-V7 of the bacterial 16S rRNA gene (Chelius and Triplett, 2001; Bodenhausen *et al.*, 2013). For multiplexed pyrosequencing (454) the 799F primer was modified at the 5‘end with a sample-specific 6mer barcode. A *Sfi*I site for ligation of 454 adaptors was added behind the barcode (5‘-GATGGCCATTACGGCCNNNNNN-799F-3’). The 1193R primer was adjusted by adding a *Sfi*I site at the 5’end for 454 ligation (5’-CCTATCCCCTGTGTGCCTTGGCAGTCGA-1193R-3’). PCRs were performed using 3 µl of DNA adjusted to 3.5 ng/µl in a total volume of 25 µl. PCR components included 1.25 U DFS-Taq DNA Polymerase (Bioron, Ludwigshafen, Germany), 1x incomplete reaction buffer, 0.3% BSA, 2 mM of MgCl_2_, 200 μM of dNTPs and 400 nM of each primer. The PCR reaction was pipetted in a laminar flow and amplified using a touch-down protocol (**Supplementary Table S8**). To minimize PCR bias 12 independent PCR reactions were performed for each DNA sample using three independent master mixes. Quality of PCR amplicons was controlled by loading 5 µl of the individual reaction on a 1% agarose gel and affirming that no band was detected in the negative control. Afterwards, the replicated PCR-reactions were combined and purified using the Agencourt AMPure XP PCR Purification kit (Beckman Coulter, Krefeld, Germany). Purified products were eluted in a final volume of 30 µl and the complete reaction loaded on a 1.5% agarose gel and ran for ~2 hours at 80 V. Bands with the correct size of ~400 bp were cut and purified using the QIAquick gel extraction kit (Qiagen, Hilden, Germany). Afterwards, the DNA concentration was determined using the PicoGreen assay as described above. 200 ng DNA of each of the barcoded amplicons were pooled in one library. To minimize the total volume of the reaction and the amount of primer-dimers, two consecutive purification steps were carried out using the Agencourt AMPure XP PCR Purification kit and the sample eluted in a final volume of 80 µl. The amplicons were then prepared for pyrosequencing as described in Bulgarelli *et al.*, 2012.

### Illumina sequencing

Amplicon libraries for Illumina sequencing were prepared in a similar manner compared to the pyrosequencing libraries to ensure comparability between the two techniques. In a first PCR the V5-V7 region of the bacterial 16S rRNA gene was amplified using the primers 799F and 1193R without adaptors and barcodes. PCR reactions were performed using 3 µl of DNA adjusted to 3.5 ng/µl in a total volume of 25 µl. PCR components include 1.25 U DFS-Taq DNA Polymerase (Bioron, Ludwigshafen, Germany), 1x incomplete reaction buffer, 0.3% BSA, 2 mM of MgCl_2_, 200 μM of dNTPs and 400 nM of each primer. To minimize PCR bias three independent PCR reactions using one master mix were prepared. The PCR reaction was pipetted in a laminar flow hood and PCR amplified (94°C/2 minutes, 94°C/30 seconds, 55°C/30 seconds, 72°C/30 seconds, 72°C/10 minutes for 25 cycles). Thereafter, single-stranded DNA was removed by adding 1 µl of Antarctic phosphatase, 1 µl Exonuclease *I* and 2.44 µl Antarctic phosphatase buffer to 20 µl of the pooled PCR product. Samples were incubated at 37°C for 30 minutes and enzymatic activity was deactivated at 85°C for 15 minutes. Samples were centrifuged for 10 minutes at 4 000 rpm and the supernatant was transferred to a new plate. 3 µl of this reaction were used for a second PCR with primers that included barcodes and Illumina adaptors. Towards this end, the 799F primer was modified at the 5‘end (5‘-AATGATACGGCGACCACCGAGATCTACACGACTGCGACTGGCGA-799F-3’). The 1193R primer was adjusted for multiplexed amplicon sequencing by adding a 12mer barcode at the 5’ end followed by the Illumina adaptor. (5’-CAAGCAGAAGACGGCATACGAGAT-NNNNNNNNNNNN-CAGCCATTTAGTGTC-1193R-3’). PCR reactions were prepared in the same way as described above using the same protocol except reducing the number of PCR-cycles to 10. The quality of PCR amplicons was controlled by loading 5 µl of all individual reactions on a 1% agarose gel, including negative controls with an expected absence of PCR products. The three independent PCR reactions were combined and loaded on a 1.5% agarose gel and the amplicons separated for approximately 2 hours at 80 V. Agarose gel pieces containing amplicons with the expected size of ~500 bp were collected with a x-tracta gel extractor (USA Scientific, Ocala, USA) and purified using the QIAquick gel extraction kit (Qiagen, Hilden, Germany). DNA concentration was determined using the PicoGreen assay as described before. 30 ng DNA of each of the barcoded amplicons were pooled thereafter in one library. A maximum of 96 samples were combined in one library. Paired-end Illumina sequencing was performed by the Max Planck Genome Center using the MiSeq sequencer generating reads with an average read length of 250 bp.

# Bioinformatic analysis of amplicon libraries

### Sequence alignment and de-multiplexing

To process the obtained raw sequencing reads for OTU picking, Illumina generated paired-end reads were first merged using the QIIME software package and the *join_paired_ends.py* script (Caporaso *et al.*, 2010). Only sequences successfully merged were included in the subsequent data analysis and all non-merged reads discarded. Approximately 82% of reads were successfully merged and used in the subsequent analyses. To de-multiplex Illumina reads, barcoded sequences were first extracted from the merged sequences using the script *extract_barcodes.py* and the output was used in the script *split_libraries_fastq.py*. Sequences that were generated with 454 were directly de-multiplexed after sequencing using the *split_libraries.py* script. Only sequences that showed no ambiguous bases, no errors in the barcode sequence, a minimum phred quality score of 27 and a minimum sequence length of 315 base pairs (bp) were retained. Afterwards, the de-multiplexed Illumina and 454 reads were concatenated for subsequent data analysis.

### OTU assignment and generation of an OTU table

OTU taxonomic assignment and generation of an OTU table was performed using the Greengenes database and the USEARCH pipeline version 7, respectively (DeSantis *et al.*, 2006; Edgar, 2013). First, de-multiplexed sequences were de-replicated in order to identify unique sequences and reduce the size of the dataset. Afterwards, OTUs were clustered using the UPARSE de-novo OTU picking algorithm and singletons discarded (Edgar, 2013). Subsequently, chimeric sequences were identified using the UCHIME algorithm (Edgar *et al.*, 2011). For OTU taxonomic assignment, representative sequences were aligned to the core set of the Greengenes database and sequences that did not match this database were removed. Afterwards, reads were mapped to their respective OTUs and an OTU table was generated that was used for further downstream analysis in R (RC Team, 2014). To avoid biases introduced by the sequencing technology, samples of the ‘soil type and environment’, ‘time course’ and ‘diversification’ experiment were separated prior to the statistical analyses and analyzed independently. If not stated otherwise, the thresholded OTU table contained only OTUs with ≥ 20 reads in at least one sample, i.e. abundant community members (ACM). The thresholded OTU table, normalized by sample size, was used for all downstream statistical analysis if not mentioned otherwise (Bulgarelli *et al.*, 2012; Schlaeppi *et al.*, 2014).

### Comparison between Illumina and 454 sequencing technologies

To test for methodological biases introduced by the used sequencing technology, a subset of 11 samples of the ‘diversification’ experiment were sequenced both with 454 and Illumina (**Table 1, Supplementary Figure S1**). While the sequencing technology did significantly affect the generated datasets (P < 0.001, PERMANOVA), this effect can be explained by a higher number of OTUs detected with Illumina compared to 454. A total of 1 967 and 4 453 OTUs were detected with 454 and Illumina, respectively. Of those, 1 919 OTUs where shared among techniques and 48 and 2 535 unique to 454 and Illumina, respectively. Additionally, we compared whether both sequencing technologies produce similar taxonomic outputs (**Supplementary Figure S2**). Phyla and families that were detected with 454, were also detected using Illumina sequencing technologies and we did not detected a taxonomic group that was absent based on the Illumina generated data but present in the 454 dataset.

### Alpha-diversity

To calculate alpha-diversity indices, all samples were corrected for the different sequencing depths of independent experimental approaches by rarefying to 2 800, 18 000 and 18 000 reads (‘soil type and environment’, ‘time course’ and ‘diversification’ experiments, respectively) using the *single_rarefaction.py* script implemented in QIIME. Afterwards, alpha-diversity indices (observed species and Shannon index) were calculated on the rarefied datasets in QIIME using the script *alpha_diversity.py*.

### CAP analysis

To calculate major determinants of community structure, a Principal Coordinate Analysis (PCoA) was conducted as previously described (Bulgarelli *et al.*, 2015). The contribution of individual factors to the observed variation in community profiles was calculated using Bray-Curtis, unweighted and weighted UniFrac distance metrics as β-diversity estimates (Beals, 1984; Lozupone and Knight, 2005).

### Defining differentially enriched OTUs

To determine OTUs enriched within the root, rhizosphere and soil compartments a similar method as described previously was used (Bulgarelli *et al.*, 2012). Statistics on the relative abundance (RA) values were performed using the *limma* package in R. For determining compartment-enriched OTUs (i.e. SoilOTUs, RhizoOTUs, RootOTUs) a linear model was fitted, using compartment as explanatory variable. Afterwards, differentially abundant OTUs were calculated using a Bayes moderated t-test (Smyth, 2005), correcting the resulting p-values using the Benjamini-Hochberg method. To detect compartment-enriched OTUs, the intersection of the corresponding pair-wise comparisons between the three compartments was calculated (P < 0.05).

To test for OTUs enriched in the root versus the soil compartments in the ‘diversification’ experiments (i.e. SoilOTUs, RootOTUs), a linear model was fitted across the comparison between the root and soil compartment. To determine the sharedOTUs more stringent statistics were applied, using Tukey’s HSD, Mann-Whitney and Bayesian statistics, according to a previously described method (Schlaeppi *et al.*, 2014). The intersection of OTUs, passing all three statistics defines the shared bacterial community (sharedOTUs).

To test for OTUs enriched across the three time points during the *A. alpina* ‘time course’ experiment, a linear model was fitted across all possible time combinations across the three different compartments (root, rhizosphere, soil). To evaluate the impact of time on the compartment-enriched OTUs a linear model was fitted across the 8 combinations between the different time points (6, 12 and 28 weeks) and the three tested compartments. To define OTUs enriched within plants residing at different developmental stages, the dataset was first separated into the different time points and then a linear model was fitted across four interaction terms, separating the two plant genotypes (*A. alpina* WT and *pep1*) from the three tested compartments.

In a similar manner, OTUs differentially enriched in the ‘soil type and environment” experiment were determined by exchanging the variable time point with the variable growth condition (FrenchSoil_ControlledConditions, FrenchSoil_NaturalSite and CologneSoil_ControlledConditions; FS-C, FS-N, CS-C). In this calculation the variable genotype included the accessions *A. alpina* Paj, Gal5 and Gal60.

### Defining a shared community within the ‘diversification’ experiment using Mann-Whitney statistics

Mann-Whitney statistics were performed on the ACM using the function *wilcox_test* in the R library *coin* as described previously (Schlaeppi *et al.*, 2014). The RootOTUs were calculated for each independent biological replicate across the different plant species based on pair-wise comparisons. For this the comparison terms “*A. thaliana*-Soil”, “*C. hirsuta*-Soil” and “*A. alpina*-Soil” were used, defining *At*OTUs, *Ch*OTUs and *Aa*OTUs, respectively (P < 0.05, FDR-corrected).

### Defining a shared community within the ‘diversification’ experiment using Bayesian statistics

Bayesian statistics were calculated using the function *BayesianIUT* in R, considering only OTUs that show a 30x higher support for the alternative hypothesis. For this the root samples of *A. thaliana*, *C. hirsuta* and *A. alpina* were compared to the group of soil samples as described previously (combining replicate experiments; Schlaeppi *et al.*, 2014).

### Ternary plots

Ternary plots were calculated on the means of the log_2_-transformed relative abundances per compartment within the respective accession, host species, time point or growth condition (indicated individually below each plot). For this a modified version of the function *ternaryplot* in the package *vcd* was used (Bulgarelli *et al.*, 2012).

### Accession numbers and scripts

The raw sequencing data were deposited in the NCBI Short Read Archive under the BioProjectID PRJNA317760, accession number SRP073035, SAMN04625632. The reference numbers for the original raw sequencing data are SRR3350817 (L1151), SRR3351958 (L1264), SRR3353796 (L1291), L35 (SRR3355061), L905 (SRR3355062) and L1118 (SRR3355063). Library details, sample information and barcode ID can be found in the **Supplementary Dataset S1**. Custom R scripts can be found at <http://www.mpipz.mpg.de/R_scripts>. This provides a downloadable folder including the used R script, input data files and custom R-packages that were separated for the individual parts of the analysis.

# References

Beals E. (1984). Bray-Curtis ordination: an effective strategy for analysis of multivariate ecological data.

Beilstein MA, Nagalingum NS, Clements MD, Manchester SR, Mathews S. (2010). Dated molecular phylogenies indicate a Miocene origin for *Arabidopsis thaliana*. *Proc Natl Acad Sci* **107**: 18724–18728.

Bodenhausen N, Horton MW, Bergelson J. (2013). Bacterial communities associated with the leaves and the roots of *Arabidopsis thaliana*. *PLoS ONE* **8**: e56329.

Bulgarelli D, Garrido-Oter R, Münch PC, Weiman A, Dröge J, Pan Y, *et al.* (2015). Structure and function of the bacterial root microbiota in wild and domesticated barley. *Cell Host Microbe* **17**: 392–403.

Bulgarelli D, Rott M, Schlaeppi K, Themaat EVL van, Ahmadinejad N, Assenza F, *et al.* (2012). Revealing structure and assembly cues for *Arabidopsis* root-inhabiting bacterial microbiota. *Nature* **488**: 91–95.

Caporaso JG, Kuczynski J, Stombaugh J, Bittinger K, Bushman FD, Costello EK, *et al.* (2010). QIIME allows analysis of high-throughput community sequencing data. *Nat Methods* **7**: 335–336.

Chelius MK, Triplett EW. (2001). The diversity of Archaea and Bacteria in association with the roots of *Zea mays* L. *Microb Ecol* **41**: 252–263.

DeSantis TZ, Hugenholtz P, Larsen N, Rojas M, Brodie EL, Keller K, *et al.* (2006). Greengenes, a Chimera-Checked 16S rRNA Gene Database and Workbench Compatible with ARB. *Appl Environ Microbiol* **72**: 5069–5072.

Edgar RC. (2013). UPARSE: highly accurate OTU sequences from microbial amplicon reads. *Nat Methods* **10**: 996–998.

Edgar RC, Haas BJ, Clemente JC, Quince C, Knight R. (2011). UCHIME improves sensitivity and speed of chimera detection. *Bioinformatics* **27**: 2194–2200.

Lozupone C, Knight R. (2005). UniFrac: a new phylogenetic method for comparing microbial communities. *Appl Environ Microbiol* **71**: 8228–8235.

RC Team. (2014). A language and environment for statistical computing. *R Foundation for Statistical Computing, Vienna*.

Schlaeppi K, Dombrowski N, Oter RG, Themaat EVL van, Schulze-Lefert P. (2014). Quantitative divergence of the bacterial root microbiota in *Arabidopsis thaliana* relatives. *Proc Natl Acad Sci* **111**: 585–592.

Smyth GK. (2005). limma: Linear models for microarray data. In: *Bioinformatics and Computational Biology Solutions Using R and Bioconductor*, Gentleman, R, Carey, VJ, Huber, W, Irizarry, RA, & Dudoit, S (eds) Statistics for Biology and Health, Springer New York, pp 397–420.

Wang R, Farrona S, Vincent C, Joecker A, Schoof H, Turck F, *et al.* (2009). PEP1 regulates perennial flowering in *Arabis alpina*. *Nature* **459**: 423–427.

# Supplementary Tables

**Supplementary Table S1: Statistical analyses of alpha-diversity estimates.**

Statistical summary of alpha-diversity indices calculated for bacterial communities found across experiments within the soil, rhizosphere and root compartment on the basis of the number of observed OTUs and Shannon index **(see also Supplementary Figure S8).** Alpha-diversity indices were calculated separately for the samples of the three experimental setups: ‘Soil type and environment’ experiments (non-thresholded dataset rarefied to 18 000 sequences); **‘**Time course’ experiment (non-thresholded dataset rarefied to 18 000 sequences): **‘**Diversification’ experiment (non-thresholded dataset rarefied to 2 800 sequences). n.s.: non-significant; * P < 0.05, ** P < 0.01, *** P < 0.001 (ANOVA).

**Supplementary Table S2: Drivers of bacterial community assembly determined using Constrained Analysis of Principal Coordinates (CAP).**

Variation (in %) between samples of the ‘soil type and environment’, ‘time course’ and ‘diversification’ experiments based on Bray-Curtis, weighted and unweighted UniFrac distances, constraining for the indicated factors. P‑value based on a permutation-based analysis of variance (PERMANOVA, 999 permutations). n.s.: non-significant, *: P < 0.05, ** P < 0.01, *** P < 0.001. CI: Confidence interval. Samples were analyzed separately for the three different experimental setups as stated in Table 1. Additionally, the ‘soil type and environment’ samples were separated as follows: ^1^Gal60 grown at the natural site and the greenhouse, ^2^Gal60, Gal5 and Paj grown in French soil in the greenhouse and ^3^Paj grown in the French and Cologne soil in the greenhouse. Same as shown in Table 2, including the used formula to calculate the variation. TP: Time point.

**Supplementary Table S3: Soil parameters.**

| **Soil** | **^1^C.org (%)** | **Clay (%)** | | **Silt (%)** | | **^2^Classification** | | | | **pH** | |  |  |
| --- | --- | --- | --- | --- | --- | --- | --- | --- | --- | --- | --- | --- | --- |
| CS Fall-10 | 4 | 21 | | 31 | | Loam | | | | 6.9 | |  |  |
| CS Spring-13 | 4 | 21 | | 31 | | Loam | | | | 6.7 | |  |  |
| CS Fall-13 (0w) | 3.5 | 21 | | 31 | | Loam | | | | 7.3 | |  |  |
| CS Fall-13 (28w) | 4 | 16 | | 31 | | Sandy loam | | | | 6.2 | |  |  |
| FS Fall-12 | 3 | 16 | | 31 | | Sandy loam | | | | 7.6 | |  |  |
| ^1^ organic carbon | | |  | |  | |  | | | |  | | |
| ^2^ Soil texture classification according to FAO | | | | | | | |  |  | | | |  |
|  | | | | | | | |  |  | | | |  |

Soil classification according to the Food and Agriculture Organization (FAO) of the United Nations. Listed are three batches of Cologne soil (CS, including harvest date) and one batch of French soil (FS, including harvest date). The batch CS Fall-13 was measured twice, before (0w) and 28 weeks (28w) after the ‘time course’ experiment. For CS Fall-13 (28w) plants were removed from the pot and the remaining soil used for soil analyses.

**Supplementary Table S4: Nutrient content of used soil types.**

| **Soil** | **Extract** | **^3^Phosphorus** | **^3^Potassium** | **^3^Magnesium** | **^3^Calcium** | **^3^Nitrate** |
| --- | --- | --- | --- | --- | --- | --- |
| CS Fall-10 | ^1^H_2_0 | 6.3 | 22.6 | 8.9 | 52.1 | 22.2 |
|  | ^2^AAE | 85.3 | 124.8 | 118.5 | 1604.1 |  |
| CS Spring-13 | ^1^H_2_0 | 1 | 11 | 5.3 | 31.8 | 12 |
|  | ^2^AAE | 20.7 | 101.9 | 144.5 | 1666.1 |  |
| CS Fall-13 (0w) | ^1^H_2_0 | 1.0 | 4.7 | 5.3 | 23.7 | 0.8 |
|  | ^2^AAE | 15.0 | 83.2 | 152.6 | 1744.2 |  |
| CS Fall-13 (28w) | ^1^H_2_0 | 1.1 | 4.6 | 7.3 | 46.4 | 1.9 |
|  | ^2^AAE | 16.8 | 83.2 | 83.2 | 1895.0 |  |
| FS Fall-12 | ^1^H_2_0 | 0.2 | 4.8 | 2 | 112.7 | 3.5 |
|  | ^2^AAE | 1.9 | 19.6 | 262.1 | 73800.8 |  |
| ^1^ determined with 1:10 (w/v) water extract as a proxy for plant-available nutrients | | | | |  |  |
| ^2^ determined with 1:10 (w/v) ammonium-acetate-EDTA (AAE) extract as a proxy for reserve nutrients | | | | | |  |
| ^3^ mg/kg |  |  | |  |  |  |
|  |  |  | |  |  |  |

Listed are three batches of Cologne soil (CS, including harvest date) and one batch of French soil (FS), including harvest date. The batch CS Fall-13 was measured twice, before (0w) and 28 weeks (28w) after the ‘time course’ experiment. For CS Fall-13 (28w) plants were removed from the pot and the remaining soil used for soil analyses.

**Supplementary Table S5: Bacterial phyla enriched in the soil, rhizosphere and root compartments across growth conditions for the ‘soil type and environment’ experiments.**

Shown are the 10 most abundant phyla detected across the 59 samples of the ‘soil type and environment’ experiment that are also depicted in **Supplementary Figure S4**. (**a)** Significant differences across growth conditions (ANOVA: aov(Phylum ~ Compartment *Habitat)). Habitat: Samples compiled based on growth conditions and soil type. (**b)** Significant differences between tested groups across compartments (Tukey’s HSD). n.s.: non-significant; * P < 0.05, ** P < 0.01, *** P < 0.001 (BH adjusted). FS_C: French soil under controlled environmental conditions. CS_C: Cologne soil under controlled environmental conditions. FS_N: French soil under native environmental conditions.

**Supplementary Table S6: Bacterial phyla enriched in the soil, rhizosphere and root compartments over time for the ‘time course’ experiment.**

Shown are the 10 most abundant phyla detected across the 106 samples from the “time course’ experiment that are also depicted in **Supplementary Figure S5**. (**a)** Significant differences across growth conditions (ANOVA: aov(Phylum ~ Compartment *Time point)). (**b)** Significant differences between tested groups across compartments (Tukey’s HSD). n.s.: non-significant; * P < 0.05, ** P < 0.01, *** P < 0.001 (BH adjusted). t1: 6 weeks, t2: 12 weeks, t3: 28 weeks.

**Supplementary Table S7: Bacterial phyla enriched in the root compartments for the ‘diversification’ experiment.**


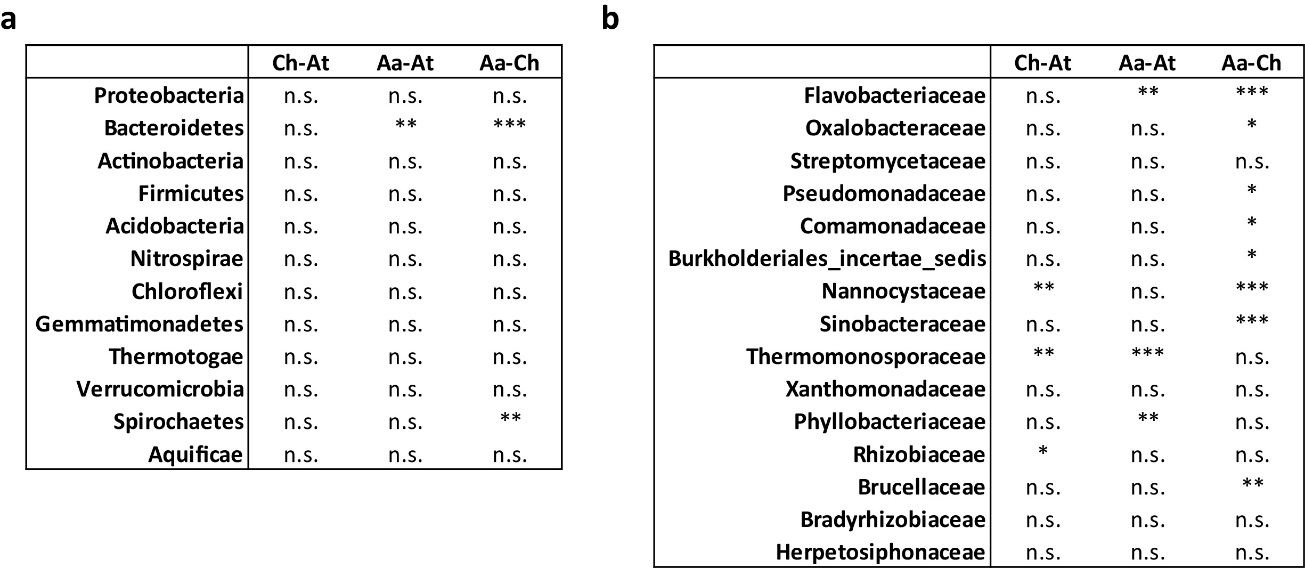


Shown are only the 12 most abundant phyla **(a)** and 15 most abundant families **(b)** detected across the 36 samples from the ‘diversification’ experiment that are also depicted in **Supplementary Figure S6**. n.s.: non-significant; * P < 0.05, ** P < 0.01, *** P < 0.001 (Tukey’s HSD, BH adjusted). Ch: *C. hirsuta*, At: *A. thaliana*, Aa: *A. alpina*.

**Supplementary Table S8**: **Touch-down PCR protocol to generate 16S rRNA gene amplicon libraries.**


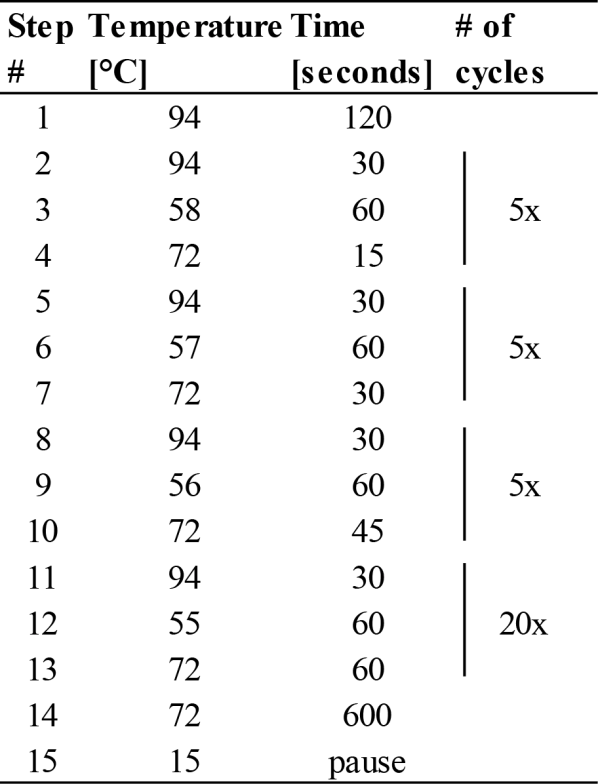


# Supplementary Figures

**Supplementary Figure S1: Methods summary.**

Methodological summary for analyzing the three experimental setups (‘soil type and environment’, ‘time course’ and ‘diversification’ experiment). Also shown are 11 samples of the ‘diversification’ experiment that were re-sequenced with Illumina sequencing technologies.

**Supplementary Figure S2: Samples from 454 and Illumina sequencing technologies co-cluster.**

Unconstrained ordination of the 201 samples from all three experimental setups. This is the same analysis as seen in Figure 2a; however, the color-coding was changed to differentiate between samples sequenced with 454 (red) or Illumina (black) protocols.

**Supplementary Figure S3: 454 and Illumina sequencing technologies produce comparable bacterial community profiles.**


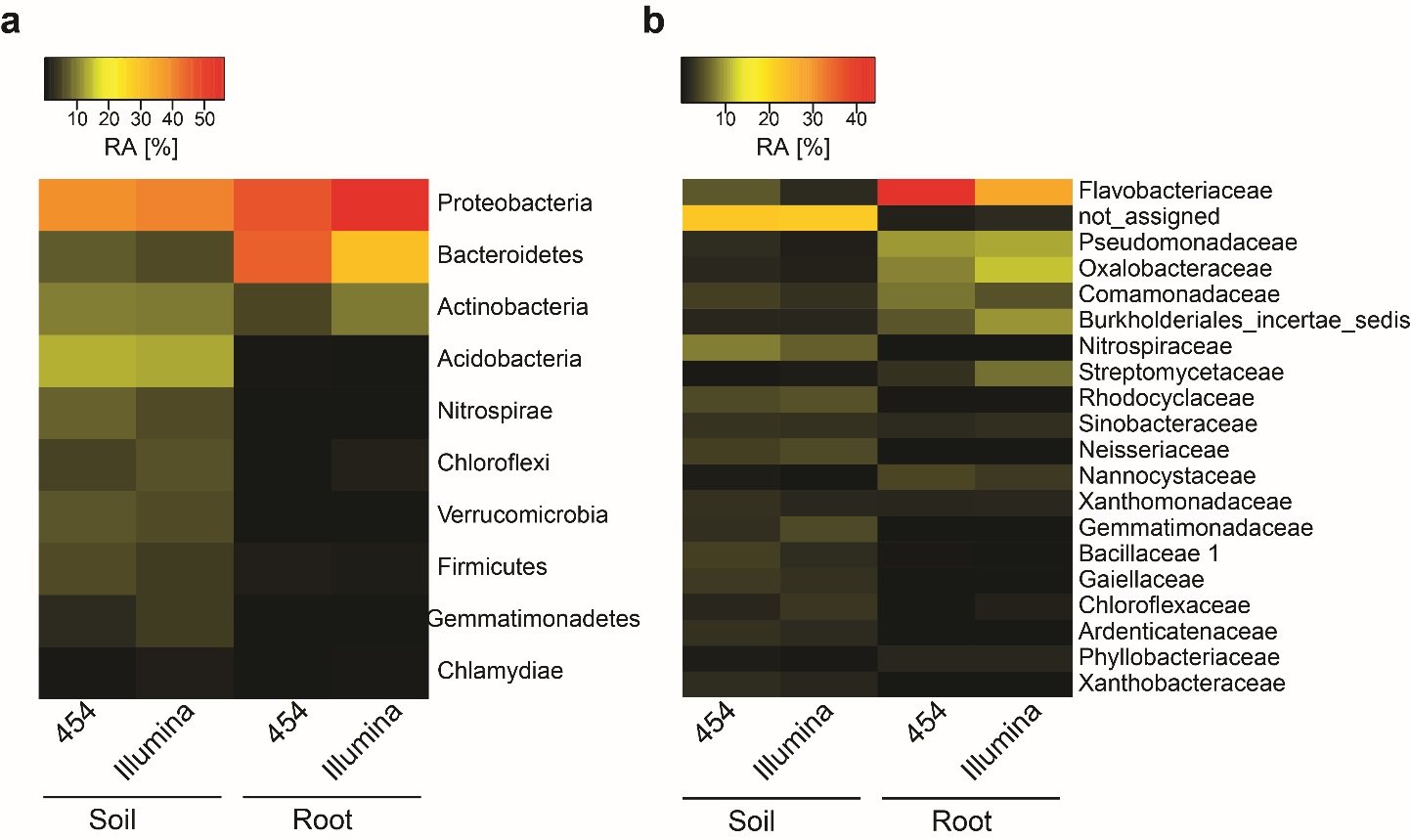


**(a,b)** Comparison between soil and root samples from the ‘diversification’ experiment (11 samples each) sequenced with 454 and Illumina protocols at phylum **(a)** and family **(b)** rank. The relative abundance (RA) of the indicated taxa is indicated in the heat map in percent.

**Supplementary Figure S4: Plant growth in different soil types and environments affects bacterial community composition in the soil, rhizosphere and root compartments of *A. alpina*.**


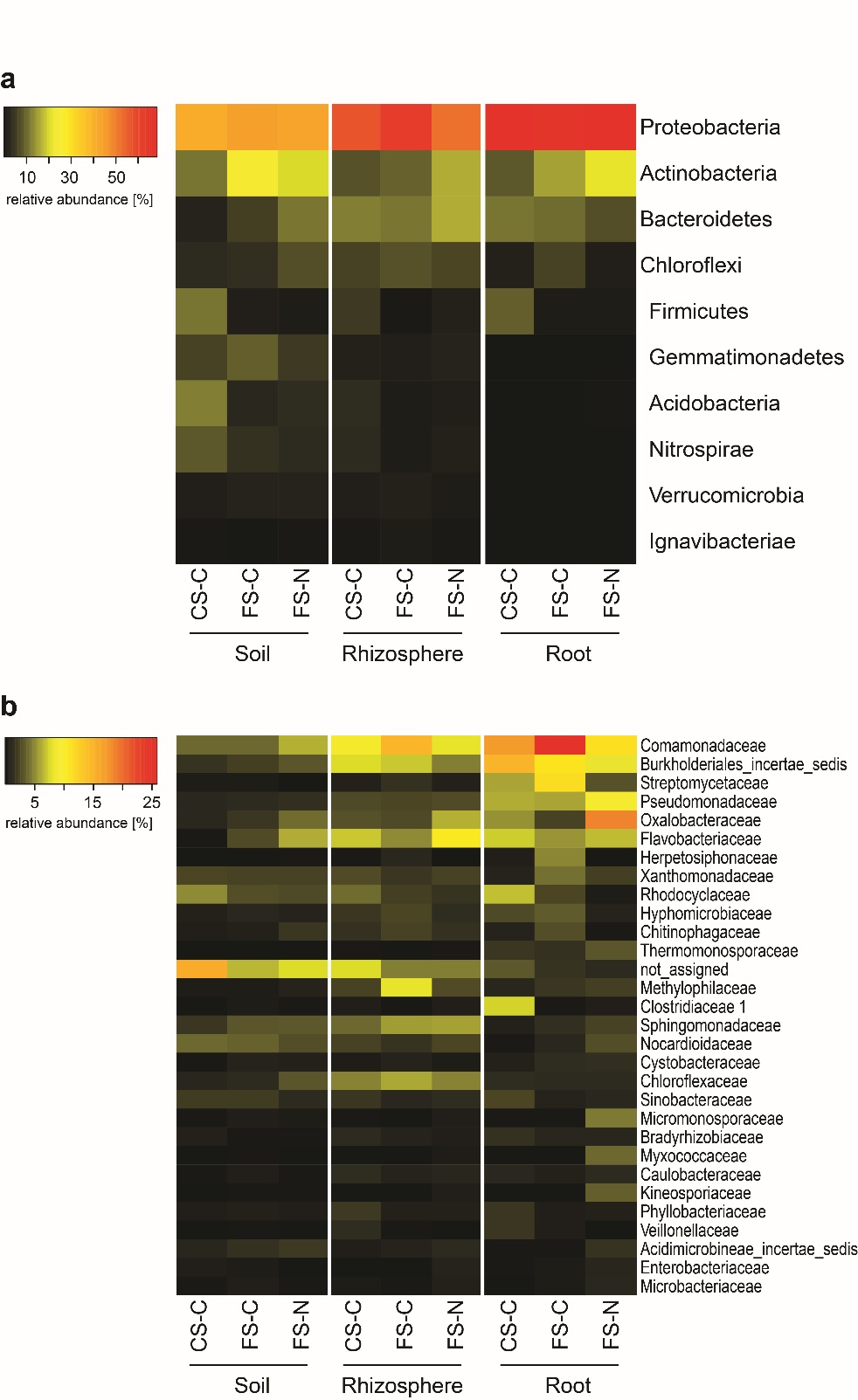


**(a)** Phylum and (**b)** family distribution of abundant bacterial taxa detected within the soil, rhizosphere and root compartments across three tested growth conditions among the 59 samples included in the ‘soil type and environment’ experiment. Growth of plants in: Cologne soil (CS) under controlled environmental conditions (C), French soil (FS) under controlled environmental conditions and French soil in natural habitats (N) in the French Alps. Depicted are the 10 most abundant phyla and 30 most abundant bacterial families.

**Supplementary Figure S5: Prolonged residence time of *A. alpina* in soil influences bacterial community composition.**


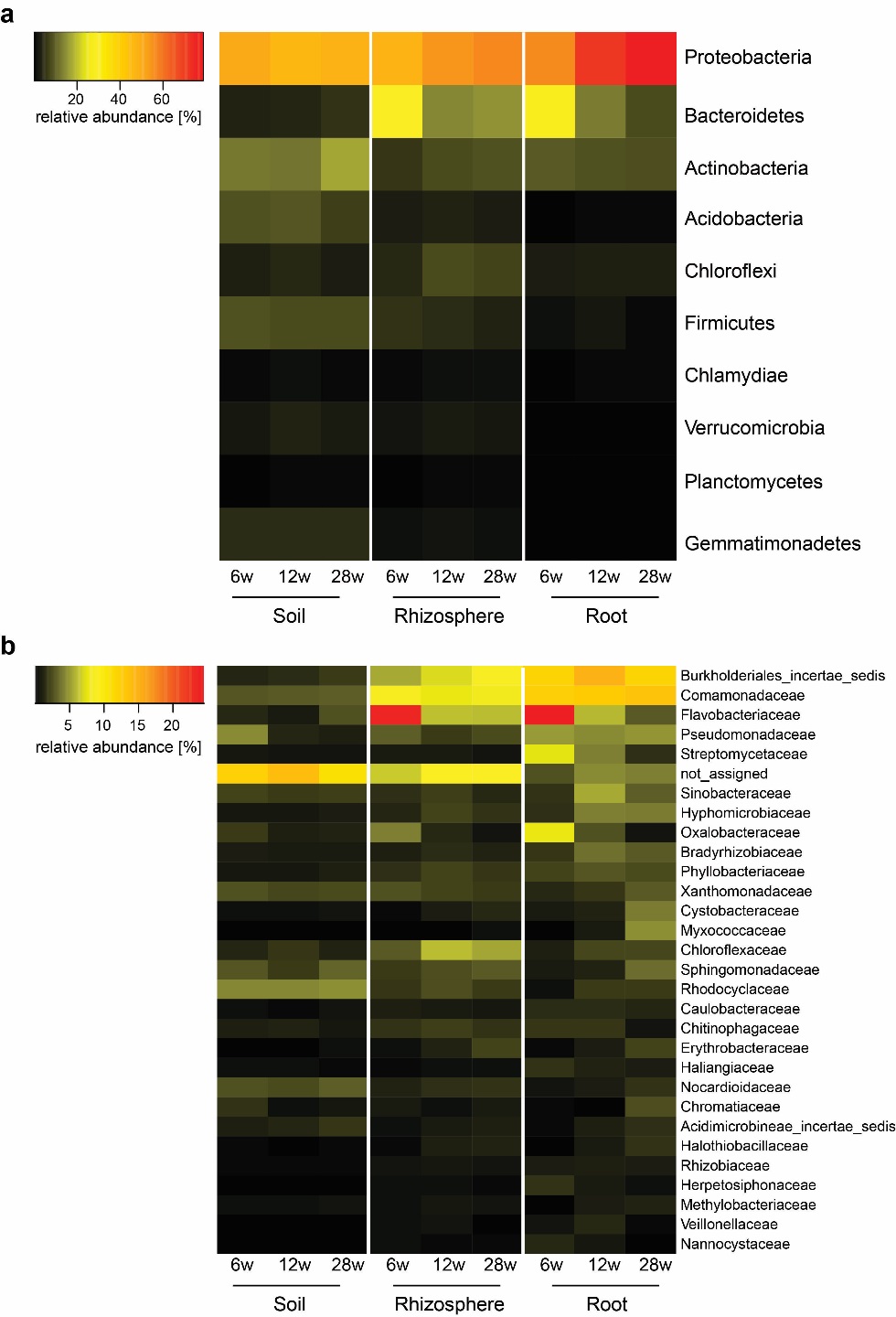


**(a)** Phylum and (**b)** family distribution of abundant bacterial taxa within the soil, rhizosphere and root compartment of *A. alpina* across the 106 samples from the ‘time course’ experiment. Bacterial community composition was monitored after 6, 12 and 28 weeks (w). The relative abundance of the 10 most abundant phyla and 30 most abundant families of the dataset are shown in percent.

**Supplementary Figure S6: Effect of time on the composition of the soil microbiota.**

**(a)** Phylum and (**b)** family distribution of abundant bacterial taxa within the soil compartment across the soil samples from the ‘time course’ experiment (see also **Supplementary Figure S5**) and separated for the two soil batches S-13 and F-13 (Spring-13 and Fall-13). Bacterial community composition was monitored after 6, 12 and 28 weeks (w). The relative abundance of the 10 most abundant phyla and 30 most abundant families of the dataset are shown in percent.

**Supplementary Figure S7: Distinct taxonomic composition of the bacterial root microbiota of *A. thaliana,* *C. hirsuta* and *A. alpina* in comparison to unplanted soil.**


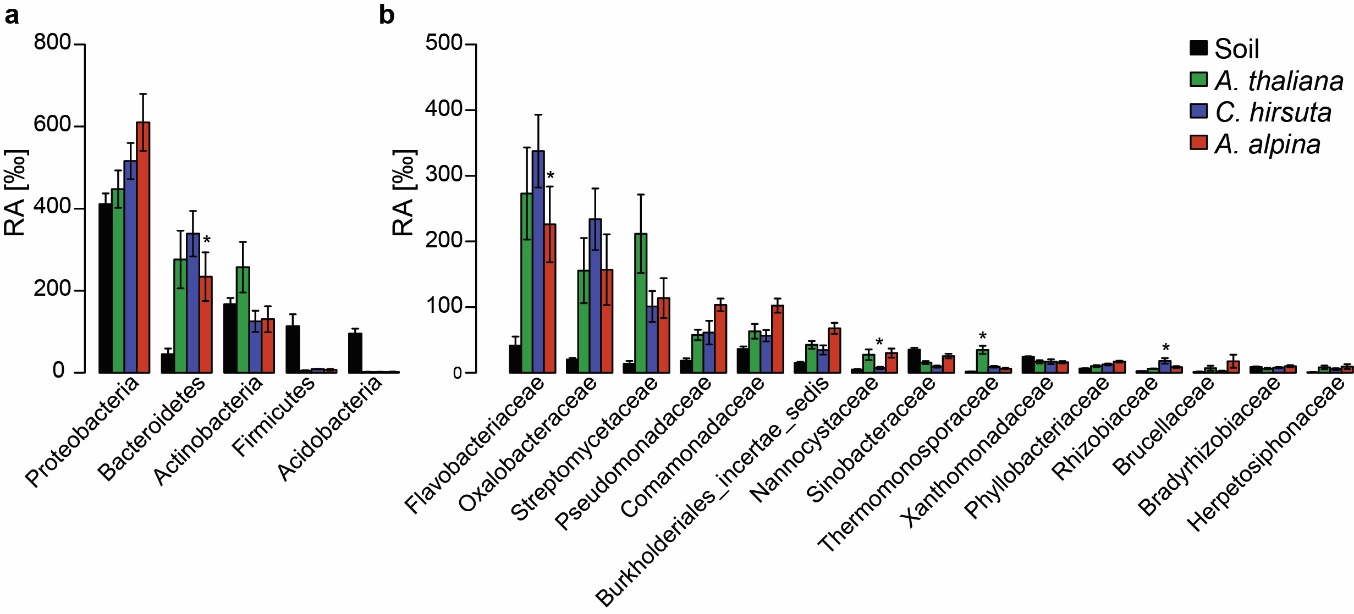


**(a)** Phylum and (**b)** family distribution of bacterial taxa detected across the 36 samples from the ‘diversification’ experiment. The bar graphs depict the relative abundance (RA) of the indicated bacterial taxa in per-mile. Soil (black), *A. thaliana* (green), *C. hirsuta* (red) and *A. alpina* (red). Error bars: standard error of the mean. Asterisk: Significant difference between *A. thaliana*, *C. hirsuta* and *A. alpina* root communities (Tukey’s HSD, P < 0.05, FDR-corrected). Depicted are the five most abundant phyla and 15 most abundant families.

**Supplementary Figure S8: The bacterial root microbiota displays a lower alpha-diversity compared to unplanted soil.**

**(a-c)** Alpha-diversity of bacterial communities in the soil, rhizosphere and root compartment calculated on the basis of the number of observed OTUs (**a-c)** and Shannon index **(d-f). (a,d)** Alpha-diversity was calculated separately for samples of the ‘soil type and environment’ experiments (non-thresholded dataset rarefied to 18 000 sequences). **b,e ‘**Time course’ experiment (non-thresholded dataset rarefied to 18 000 sequences). (**c,f) ‘**Diversification’ experiment (non-thresholded dataset rarefied to 2 800 sequences). Significance values based on P < 0.001 (Tukey’s HSD). *At*: *A. thaliana*, *Ch*: *C. hirsuta*, *Aa*: *A. alpina* (Aa), BS: Bulk soil, Rh: Rhizosphere, R: Root. CC: Cologne soil under controlled environmental conditions. FC: French soil under controlled environmental conditions. FN: French soil under native environmental conditions.

**Supplementary Figure S9: Constrained principal coordinates analysis for interacting factors in the ‘soil type and environment’ and ‘time course’ experiment.**

Detected variation (in %) across samples of the ‘soil type and environment’ **(a)** and ‘time course’ **(b)** experiment based on the Bray-Curtis distance, constraining for two different factors. P-value based on a permutation-based analysis of variance (PERMANOVA, 999 permutations). *** P < 0.001. CI: Confidence interval. Used formulas: Soil * Environment + Condition (Compartment) **(a)** and Compartment * Timepoint + Condition (Soil) **(b)**. TP: Time point.

**Supplementary Figure S10: *A. alpina* exerts a strong influence on root and rhizosphere communities regardless of growth conditions.**


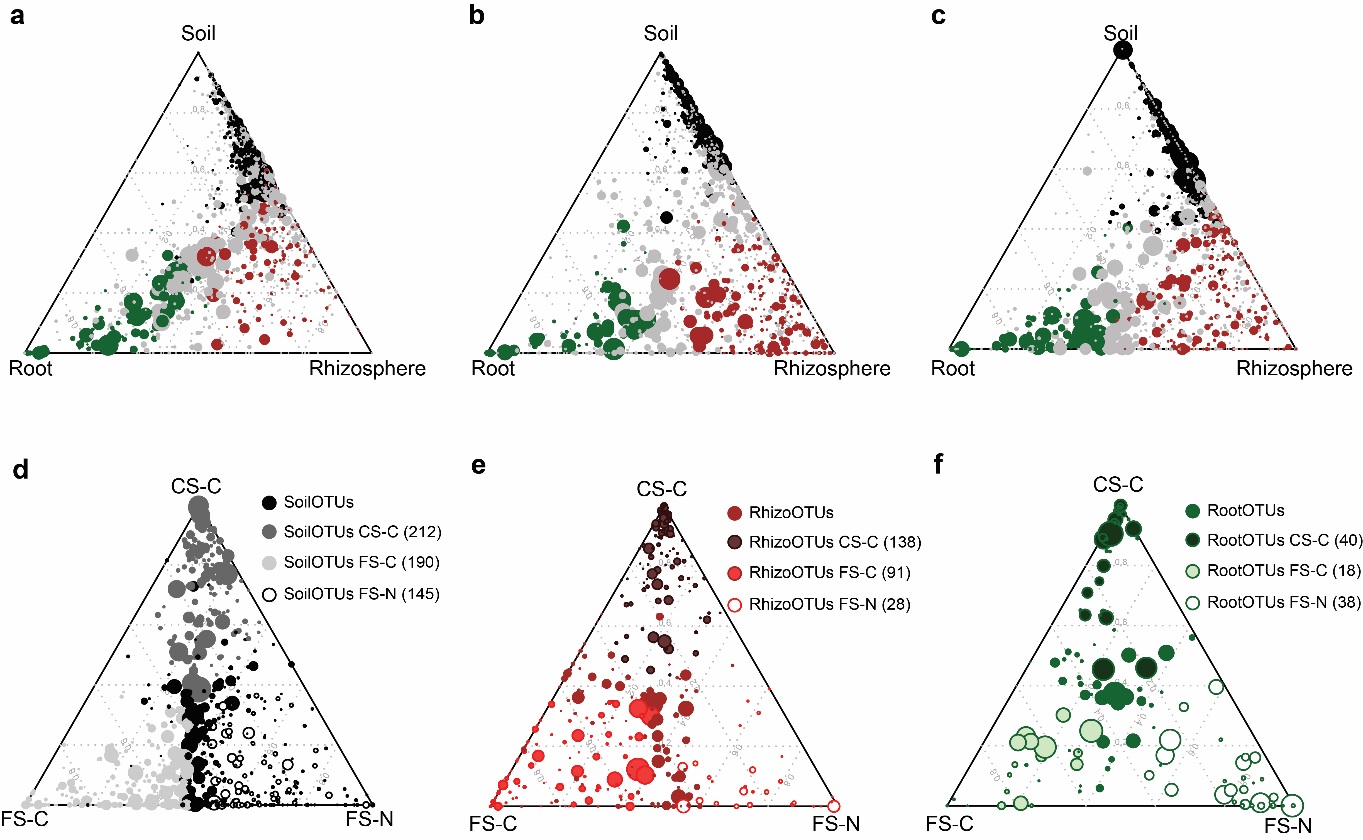


**(a-c)** Soil‑, rhizosphere‑ and root‑enriched OTUs detected across the 59 samples of the ‘soil type and environment’ experiment, growing *A. alpina* in French soil under natural environmental conditions **(**FS-N; **a)**, in the French soil under controlled environmental conditions **(**FS-C; **b)** or in the Cologne soil under controlled environmental conditions **(**CS-N; **c)**. (**d-f)** OTUs enriched under different growth conditions in the soil (black), rhizosphere (brown) and root (green) compartment, respectively. Each circle depicts one OTU and the size of the circle reflects its relative abundance (RA). The position of each circle is determined by the relative contribution(s) of the indicated compartments to the RA. The dotted grids and numbers inside the plot represent 20% increments of contribution from each compartment. Growth condition-dependent SoilOTUs (d), RhizoOTUs (e) and RootOTUs (f). Number in brackets indicate enriched OTUs based on a Bayesian moderated t-test; P < 0.05 (FDR-corrected).

**Supplementary Figure S11: Evolutionary relatedness and sonication of *A. alpina*, *C. hirsuta* and *A. thaliana* roots.**

**
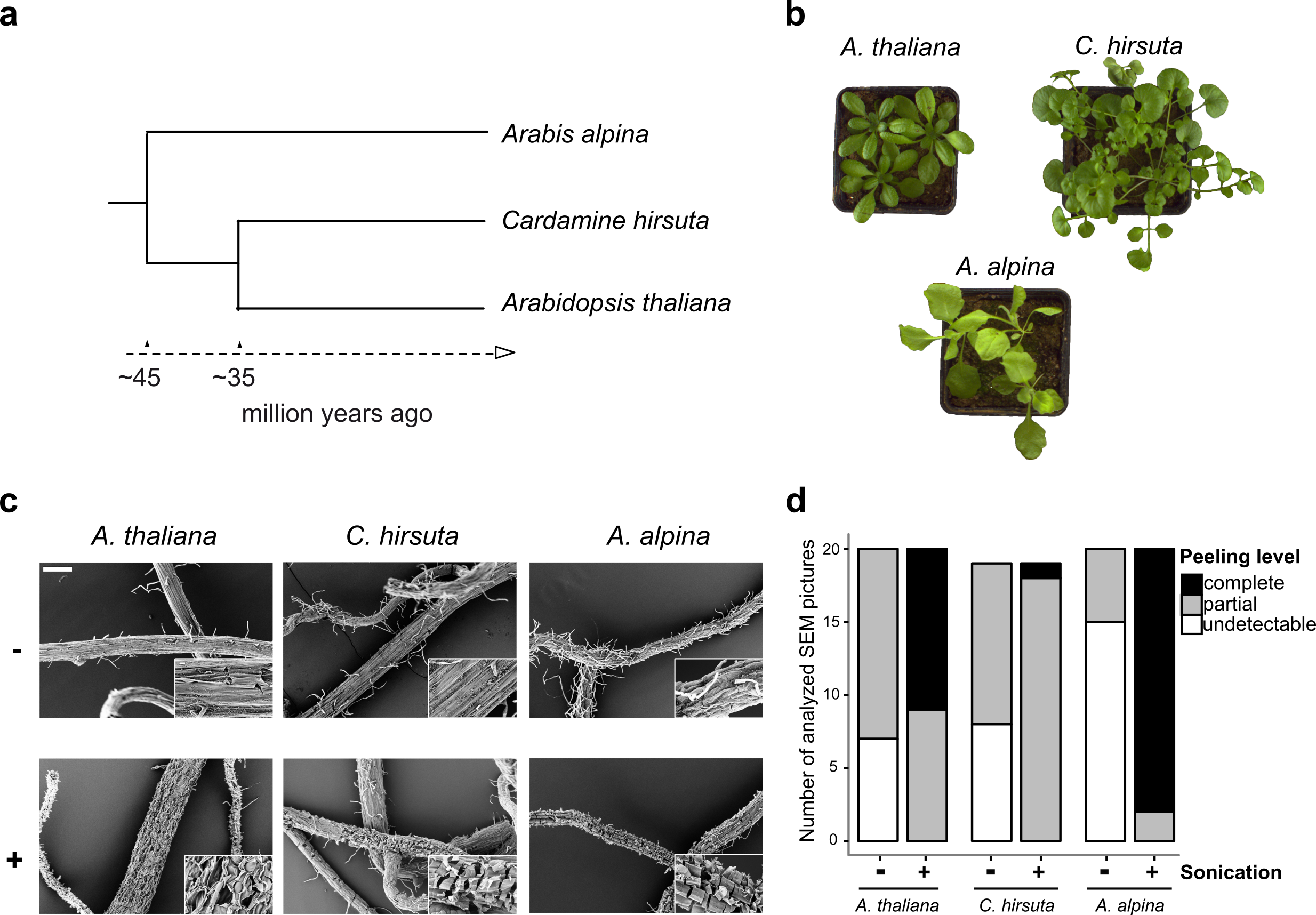
**

**(a)** Evolutionary relatedness of. *A. alpina*, *C. hirsuta* and *A. thaliana.* Divergence time estimates indicated below the phylogenetic tree are based on the NADH dehydrogenase subunit F and phytochrome A sequences (Beilstein *et al.*, 2010). (**b)** Growth phenotype of *A. alpina*, *C. hirsuta* and *A. thaliana* grown in Cologne soil for 6 weeks. (**c)** Representative pictures of roots from *A. thaliana*, *C. hirsuta* and *A. alpina* with (+) and without sonication (-) using scanning electron microscopy (SEM). Bar = 100 Micrometer. Inset pictures = 10x magnification. (**d)** Semi-quantitative scoring of root epidermal peeling with (+) and without (-) sonication treatment. A total of 20 pictures from two roots were analyzed per plant species and treatment. Peeling levels were estimated by scoring roots for ‘undetectable’ (white), ‘partial’ (grey) or ‘complete’ (black) peelings.

**Supplementary Figure S12: A shared bacterial root microbiota assembles on *A. thaliana*, *C. hirsuta* and *A. alpina*.**


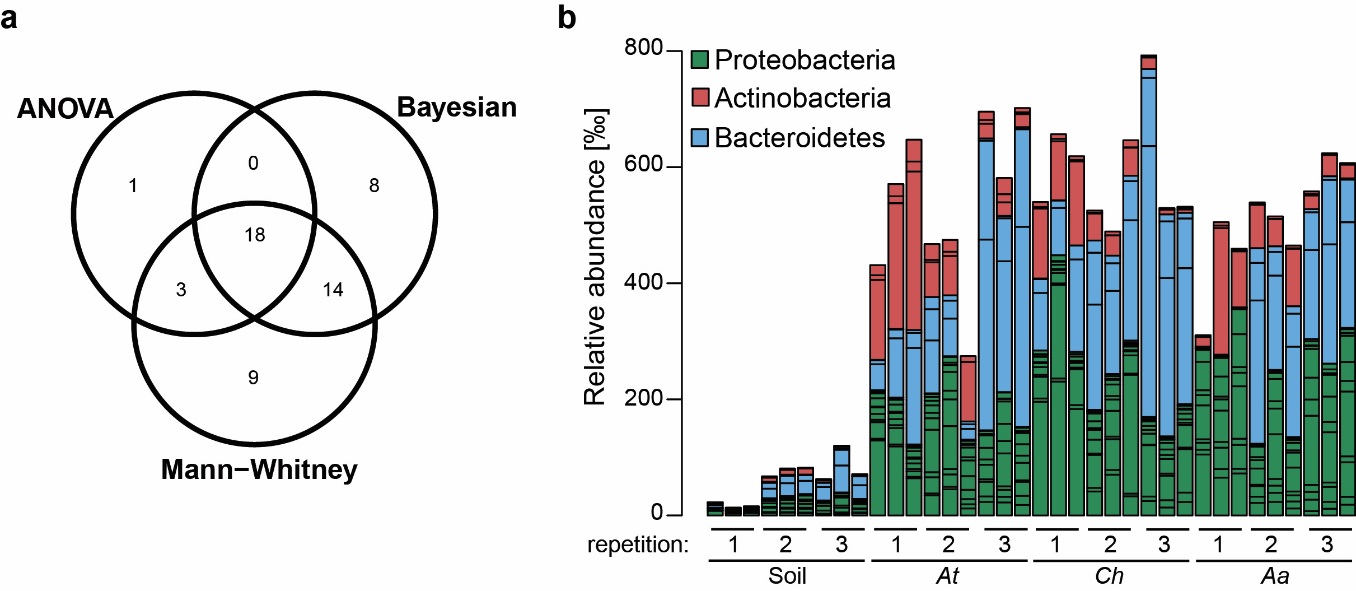


**(a)** Venn diagram depicting the total number of RootOTUs detected across the 36 samples of the ‘diversification’ experiment that were shared between *A. thaliana* (At), *C. hirsuta* (Ch) and *A. alpina* (Aa) based on parametric Tukey’s HSD, Bayesian and parametric Mann-Whitney statistics across three independent biological replicates. A shared bacterial community of 18 OTUs was defined (sharedOTUs). (**b)** Stacked relative abundance (RA) in per-mile of sharedOTUs across three independent biological replicates. Each segment corresponds to one of the sharedOTUs. Proteobacteria (green), Actinobacteria (red) and Bacteroidetes (blue).
